# Association of Habitual Patterns and Types of Physical Activity and Inactivity with MRI-Determined Total Volumes of Visceral and Subcutaneous Abdominal Adipose Tissue in a General White Population

**DOI:** 10.1371/journal.pone.0143925

**Published:** 2015-11-30

**Authors:** Karina Fischer, Daniela Rüttgers, Hans-Peter Müller, Gunnar Jacobs, Jan Kassubek, Wolfgang Lieb, Ute Nöthlings

**Affiliations:** 1 Department of Nutrition and Food Sciences, University of Bonn, Bonn, Germany; 2 Department of Neurology, University of Ulm, Ulm, Germany; 3 PopGen Biobank, University Hospital Schleswig-Holstein, Kiel, Germany; 4 Institute of Epidemiology, Kiel University, Kiel, Germany; 5 Department of Geriatrics, University Hospital Zurich & Centre on Aging and Mobility, University of Zurich, Zurich, Switzerland; University of Leipzig, GERMANY

## Abstract

Population-based evidence for the role of habitual physical activity (PA) in the accumulation of visceral (VAT) and subcutaneous (SAAT) abdominal adipose tissue is limited. We investigated if usual patterns and types of self-reported PA and inactivity were associated with VAT and SAAT in a general white population. Total volumes of VAT and SAAT were quantified by magnetic resonance imaging in 583 men and women (61 ± 11.9 y; BMI 27.2 ± 4.4 kg/m^2^). Past-year PA and inactivity were self-reported by questionnaire. Exploratory activity patterns (APAT) were derived by principal components analysis. Cross-sectional associations between individual activities, total PA in terms of metabolic equivalents (PA MET), or overall APAT and either VAT or SAAT were analyzed by multivariable-adjusted robust or generalized linear regression models. Whereas vigorous-intensity PA (VPA) was negatively associated with both VAT and SAAT, associations between total PA MET, moderate-intensity PA (MPA), or inactivity and VAT and/or SAAT depended on sex. There was also evidence of a threshold effect in some of these relationships. Total PA MET was more strongly associated with VAT in men (*B* = -3.3 ± 1.4; *P* = 0.02) than women (*B* = -2.1 ± 1.1; *P* = 0.07), but was more strongly associated with SAAT in women (*B* = -5.7 ± 2.5; *P* = 0.05) than men (*B* = -1.7 ± 1.6; *P* = 0.3). Men (-1.52 dm^3^ or -1.89 dm^3^) and women (-1.15 dm^3^ or -2.61 dm^3^) in the highest (>6.8 h/wk VPA) or second (4.0–6.8 h/wk VPA) tertile of an APAT rich in VPA, had lower VAT and SAAT, respectively, than those in the lowest (<4.0 h/wk VPA) tertile (*P* ≤ 0.016; *P*_trend_ ≤ 0.0005). They also had lower VAT and SAAT than those with APAT rich in MPA and/or inactivity only. In conclusion, our results suggest that in white populations, habitual APAT rich in MPA might be insufficient to impact on accumulation of VAT or SAAT. APAT including ≥4.0–6.8 h/wk VPA, by contrast, are more strongly associated with lower VAT and SAAT.

## Introduction

Accumulation of abdominal adipose tissue (AAT) has been considered a consequence of modern lifestyle patterns, including habitual physical activity (PA) and inactivity (IA) [1, 2]. Both excess visceral (VAT) and subcutaneous (SAAT) AAT have recently been claimed to play a role in cardiometabolic disease etiology [1–3]. Whereas VAT has been shown to contribute to systemic inflammation [1, 4] and has been suggested to be the key causal driver of the cardiovascular risk associated with the metabolic syndrome [1, 4], the impact of SAAT on chronic diseases is less well understood [3–5].

A number of epidemiological studies have observed associations between PA [6–8] and/or IA [9–11] and simple anthropometric measures, such as waist circumference and waist-to-hip ratio as surrogate measures for total AAT. These measures correlate well with direct measurements of total AAT [12], but are unable to distinguish between VAT and SAAT [13, 14]. In contrast, specific amounts of VAT and SAAT can be directly measured by non-invasive imaging techniques such as magnetic resonance imaging (MRI) and computed tomography (CT) [15]. However, as the availability of imaging devices for the use in large-scale studies is limited [15], only a few population-based studies have used these techniques. Moreover, studies that did use imaging techniques usually applied single- or few-slice MRI or CT scans [16–18], which, as opposed to volumetric multi-slice or whole-body scans, do not cover the whole abdomen and, therefore, provide less accurate estimates of total volumes of VAT and SAAT [15, 19, 20].

Apart from several intervention studies [21–25] or population-based studies in specific population groups including women [26–32], men [33], adolescents [34], or children [35], to our knowledge only four studies on PA and directly measured VAT and/or SAAT were conducted in an adult population of both sexes using either single-slice CT [16, 17] or few- [18] and multi-slice [36] MRI, but not whole-abdomen scans. Moreover, whereas intervention studies have demonstrated that both the volume [25, 37] and intensity [25] of exercise or even light PA [38] can reduce VAT and SAAT in the short term, epidemiological evidence for the role of habitual activity patterns (APAT) or individual types of PA and IA in long-term accumulation of VAT and SAAT is limited. Importantly, previous studies have not investigated APAT in relation to AAT that integrate the interrelations and synergy effects of multiple aspects of PA and IA [39]. The aim of the present study was to explore if, and to what extent, overall APAT and individual types of habitual PA and IA were associated with MRI-determined total volumes of VAT and SAAT in a general white population. Moreover, we investigated whether these associations were independent of overall obesity, or modified by sex, age, and body mass index (BMI).

## Materials and Methods

### Participants and study design

Participants (*n* = 583; 59% men; 61 ± 11.9 y; BMI 27.2 ± 4.4 kg/m^2^) aged 25–83 years were recruited from the PopGen control cohort, which is a sample of 1316 individuals of the general population of Kiel, Northern Germany, recruited into the PopGen biobank as controls for case-control studies [40, 41]. Of the random sample of 23,000 local residents who were identified through official population registries and invited to participate in the study, 4,267 individuals (19%) agreed to be part of the PopGen control population. There were no specific characteristics of the non-responders in comparison to responders and the wider background population. Of the 4,267 individuals comprising the PopGen control population, 747 participants (18%) also agreed to further participate in the follow-up of the newly established control cohort. Together with 569 blood donors recruited by the University Medical Center Schleswig-Holstein in Kiel, Germany, these two groups constituted the 1316 individuals of the final PopGen control cohort. Baseline examinations of the cohort were carried out from 2005 to 2007 and comprised a questionnaire with sociodemographic and sociocultural questions as well as questions on medical history, family history of disease, prevalent cardiometabolic and other chronic diseases, use of medication and drugs, and lifestyle factors such as smoking status and alcohol consumption, anthropometric and medical examinations, blood sampling, and analyses of a range of biomarkers [41]. Between 2010 and 2012, a total of 930 cohort participants agreed to take part in the first follow-up that included questionnaires on medical history and demographic, lifestyle, and health-related characteristics such as diet, PA and IA, as well as physical examinations and bio sampling [41]. Of these subjects, 653 individuals with complete PA, IA, and dietary data agreed to undergo whole-body MRI to measure body fat distribution. From these, subjects who may have over- (>16.7 MJ [4000 kcal]/d women; >17.6 MJ [4200 kcal]/d men) or under- (<2.5 MJ [600 kcal]/d) reported their energy intakes [42] (*n* = 9) as well as subjects with incomplete data on MRI variables (*n* = 58) or any covariate of interest (*n* = 5) were excluded. After exclusions, a total of 583 individuals were included in the present cross-sectional study.

### Ethics statement

The study protocol was in accordance with the standards for the use of human subjects in research as outlined in the Declaration of Helsinki and was approved by the ethics committee of the Medical Faculty of the University of Kiel, Germany. All participants gave their written informed consent to the study.

### Assessment of habitual PA and IA

Habitual PA and IA were assessed using questions from a short, self-administered questionnaire used in the European Prospective Investigation into Cancer and Nutrition (EPIC) study [43] to estimate the average number of hours per week spent on common individual PA and IA over the past year. Contrary to the original questionnaire that was validated against repeated objective measures of fitness and energy expenditure [43], in the version of the questionnaire used in the present study, individual PA and IA asked were not specifically divided into occupational and recreational activities and the questionnaire was not revalidated. This simplification was done to reduce the burden of the participants who had to complete a number of different questionnaires during the first follow-up examination. Participants were asked to report the duration and frequency of time spent walking, cycling, engaging in sports, and gardening (all separately for summer and winter seasons, of which the average was calculated for analysis) as well as on household work and manual/do-it-yourself (DIY) work. In addition, subjects reported the number of flights of stairs climbed per day. The duration of stair climbing per week was calculated with the assumption of 20 steps/flight and that, on average, 72 steps/min were climbed [44]. In addition, participants were asked to report how many hours per day they spent watching TV and sleeping (average of sleep during the day and at night). Total PA reported (h/d) was calculated as the sum of reported time spent on housework, walking, gardening, DIY work, stair climbing, sports, and cycling. Total IA reported (h/d) was calculated as the sum of reported time spent sleeping and watching TV. Time that was not reported was assumed to be spent at rest [31]. Assumed overall 24-h IA (h/d) was calculated as the difference between 24 hours and total PA reported.

According to metabolic equivalents of tasks (METs) [45], individual activities were classified into four categories of intensity: 1) IA (<1.5 METs); 2) light-intensity PA (1.6–2.9 METs); 3) moderate-intensity PA (MPA; 3.0–6.0 METs); and 4) vigorous-intensity PA (VPA; >6.0 METs). To express the sum of all PA in terms of total MET-h/wk (PA MET) [45], hours per week spent on each PA were multiplied by the corresponding MET (in parentheses) [45] and summed for all individual PA: i.e. housework (3.5), walking (3.5), gardening (3.8), DIY work (4.5), stair climbing (4.0), sports (6.0), and cycling (6.8). Both sleeping (0.95) and watching TV (1.3) were considered as reported IA and thus were not included in the PA MET score. For a reference adult, one MET is equal to 1 kcal expended per kg body weight per hour of sitting at rest [45].

### Assessment of covariates

Assessment of past-year dietary and energy intakes by an evaluated 112-item food-frequency questionnaire (FFQ) was described previously [46]. This FFQ was based on a validated FFQ used in the baseline assessment of the European Prospective Investigation into Cancer and Nutrition (EPIC)-Potsdam study. It was specifically designed for the follow-up of the EPIC-Potsdam study and adapted to yield high response rates and complete FFQ data, and to comprise particularly those food items that most discriminated participants with regard to food and nutrient intake.

Information on subject characteristics including age, sex, and smoking status was collected by self-administered questionnaires. Anthropometric and blood pressure measurements as well as biochemical analyses were described elsewhere [46].

### Adipose tissue assessment

Analysis of whole-body MRI data [47] focusing on the abdominal region (from the top of the liver to the femur heads [13]) was performed by ATLAS (Automatic Tissue Labelling Analysis Software; inhouse-developed at the Department of Neurology, University of Ulm, Ulm, Germany) [14] segmenting total volumes of AAT into VAT and SAAT [14, 48] as described previously [46]. In short, after merging individual cross-sectional MRI volumes of defined body parts to a continuous three-dimensional dataset, the segmentation of AAT into VAT and SAAT was performed using the ARTIS (Adapted Rendering for Tissue Intensity Segmentation) algorithm. SAAT was defined as the sum of all AAT voxels underneath the skin layer surrounding the abdomen from the top of the liver to the femur heads. VAT was defined as the sum of all AAT voxels inside the abdominal muscular wall. Liver fat and fat in the intestinal loops as well as minor MRI artifacts (mainly caused by stents or hip implants) were manually excluded from segmented VAT during post-processing, whereas participants with major MRI artifacts were excluded from further analysis. All ATLAS-based analyses were performed by the same observer to minimize interrater variability. The accuracy of the semi-automatically ATLAS determined volumes of VAT and SAAT was validated against manually determined AAT volumes using the image analysis software slice-O-matic (version 4.2, Tomovision, Montreal, Canada) in a subset of 38 participants [49]. For both VAT (r = 0.996) and SAAT (r = 0.996), AAT volumes analyzed by ATLAS and slice-O-matic yielded high intraclass correlations.

### Statistical analysis

Statistical analysis was performed using SAS version 9.3 (SAS Institute, Inc., Cary, North Carolina, USA). Differences in subject characteristics between men and women or subjects with low total PA (MET-h/wk < 96.1) and high total PA (MET-h/wk ≥ 96.1) were analyzed using a *χ* ^*2*^ test for categorical and Student’s *t* test for continuous variables. For Student’s *t* test and linear regression analysis, distributions of continuous variables were examined and, if necessary, transformed to reach or (in the case of several activity variables) approach normality examined by skew, kurtosis, and Kolmogorov-Smirnov test.

Principal components analysis (PCA) with varimax orthogonal rotation on nine activity items (housework, walking, gardening, DIY work, stair climbing, sports, cycling, sleeping, and watching TV) was used to derive independent APAT variables that maximally explain the variation among the nine activity items. Principal components that met both the eigenvalue ≥1.0 criterion and Scree test were chosen to be representative of independent APAT, and the variation in the activity items explained by each APAT was determined.

Because the residuals of several activity variables were not normally distributed, even after transformation, robust multiple linear regression with MM estimation [50] was used to investigate associations between individual activity or APAT variables and VAT and SAAT. All models were adjusted for sex (except sex strata), age (except age strata), total energy intake, and smoking status (never [smoking period <3 mo], former [smoking period ≥3 mo], and current smoker), and either BMI (except BMI strata) (model 1) or height (model 2) to account for overall adiposity [51] or body size, respectively. As BMI was highly correlated with both VAT (r = 0.70) and SAAT (r = 0.80) [46], model 2 was considered the more appropriate and final model. Potential effect modification by sex, median age (<62.0 and ≥62.0 y), or median BMI (<26.7 and ≥26.7 kg/m^2^) was investigated by including a multiplicative interaction term in the models. Regression coefficients (*B*) and standard errors (SE) are reported for unstandardized variables. Generalized linear models (ANCOVA) adjusted for age, total energy intake, smoking status, and height (model 2) were used to calculate adjusted least-squares means for VAT and SAAT by sex and non-sex-specific tertile of individual activity or APAT variables. Statistical differences in unadjusted and adjusted mean VAT and SAAT values between tertiles were corrected for multiple comparisons by using the Tukey-Kramer procedure. Linear regression analysis was performed to test for a linear or quadratic trend across the tertiles by treating the median values of each category as continuous variables.

Reported *P* values are two-sided. *P* < 0.05 was considered statistically significant unless otherwise indicated. To account for multiple testing of 38 subject characteristics and 14 or 13 activity and APAT variables under possible dependence, the Benjamini-Yekutieli (B-Y) false discovery rate (FDR) [52] yielding significance thresholds of *P* ≤ 0.012, *P* ≤ 0.015, and *P* ≤ 0.016, respectively, was applied (critical *P* = α/Σ (1/k) where *α* = 0.05 and *k* = 38, 14, or 13 tests).

## Results

### Subject characteristics

Subject characteristics are presented by sex and non-sex-specific median PA MET (Table 1). Considering B-Y FDR significance (*P* ≤ 0.012), men with higher PA levels (≥96.1 MET) had lower plasma concentrations of C-reactive protein, higher intakes of dietary fiber, spent more hours on all types of reported PA and less hours on assumed overall 24-h IA, and had lower volumes of SAAT and subcutaneous trunk adipose tissue, but not VAT (*P* = 0.2), than men with lower PA levels (<96.1 MET). By contrast, women with higher PA levels (≥96.1 MET) were older, had a lower ratio of intakes of n-6 to n-3 fatty acids and higher intakes of dietary fiber, spent more hours on all types of PA reported (except for stair climbing; *P* = 0.04) and less hours on assumed overall 24-h IA, but did not have B-Y FDR significantly lower volumes of SAAT and subcutaneous trunk adipose tissue (both *P* = 0.026) or VAT (*P* = 0.3) than women with lower PA levels (<96.1 MET). Subject characteristics were also calculated for tertiles of three APAT (S1 Table) described below.

**Table 1:** Characteristics of white adult study subjects by sex and level of reported total physical activity.^a^

Characteristics	Men			Women			Total population
	PA < 96.1 MET-h/wk	PA ≥ 96.1 MET-h/wk	Total men	PA < 96.1 MET-h/wk	PA ≥ 96.1 MET-h/wk	Total women	
Subjects [*n* (%)]	194 (57)	148 (43)	342 (59)	97 (40)	144 (60)	241 (41)	583
***Anthropometric parameters***							
Age (y)	60.2 ± 12.1	63 ± 9.3	61.4 ± 11.0	57.8 ± 13.7	62.1 ± 12.3^b^	60.4 ± 13.0	61.0 ± 11.9
Weight (kg)	87.5 ± 13.7	85.6 ± 13.0	86.7 ± 13.4	74.3 ± 17.4	70.2 ± 12.7	71.9 ± 14.9^c^	80.5 ± 15.8
Height (cm)	177.8 ± 7.6	177.4 ± 6.8	177.6 ± 7.3	163.7 ± 7.8	163.3 ± 7.0	163.5 ± 7.3^c^	171.8 ± 10.1
BMI (kg/m^2^) ^d^	27.7 ± 3.8	27.2 ± 3.5	27.4 ± 3.7	27.7 ± 5.8	26.4 ± 4.8	26.9 ± 5.3	27.2 ± 4.4
Waist circumference (cm)	101.1 ± 11.0	99.2 ± 10.8	100.3 ± 11.0	91.9 ± 14.4	89.0 ± 12.5	90.2 ± 13.3^c^	96.1 ± 13.0
Waist-to-hip ratio	1.0 ± 0.1	1.0 ± 0.1	1.0 ± 0.1	0.9 ± 0.1	0.9 ± 0.1	0.9 ± 0.1^c^	0.9 ± 0.1
Waist-to-height ratio	0.6 ± 0.1	0.6 ± 0.1	0.6 ± 0.1	0.6 ± 0.1	0.5 ± 0.1	0.6 ± 0.1	0.6 ± 0.1
***Biochemical parameters***							
Systolic blood pressure (mmHg)	141.8 ± 16.8	142.2 ± 20.1	142.0 ± 18.2	134.8 ± 16.9	137.0 ± 17.9	136.2 ± 17.5^c^	139.6 ± 18.2
Diastolic blood pressure (mmHg)	85.7 ± 8.4	86.3 ± 9.2	86.0 ± 8.7	83.5 ± 9.5	83.9 ± 8.1	83.7 ± 8.7^c^	85.1 ± 8.8
Plasma C-reactive protein (mg/L) ^d^	5.5 ± 10.4	2.3 ± 2.0^b^	4.3 ± 8.4	4.2 ± 6.2	2.8 ± 2.3	3.4 ± 4.4	3.9 ± 7.0
Whole blood HbA1c (%) ^d^	5.7 ± 0.6	5.8 ± 0.7	5.7 ± 0.6	5.7 ± 0.5	5.7 ± 0.5	5.7 ± 0.5	5.7 ± 0.6
Total plasma cholesterol (mg/dL)	217.9 ± 38.7	219.5 ± 43.4	218.6 ± 40.7	226.9 ± 42.7	235.1 ± 41.9	231.8 ± 42.3^c^	224.0 ± 41.9
Plasma HDL cholesterol (mg/dL)	59.1 ± 15.2	59.3 ± 15.2	59.2 ± 15.2	72.6 ± 19.0	77.2 ± 19.0	75.4 ± 19.1^c^	65.8 ± 18.7
Plasma LDL cholesterol (mg/dL)	131.6 ± 32.9	132.8 ± 35.6	132.1 ± 34.0	129.9 ± 35.4	132.6 ± 34.5	131.5 ± 34.8	131.9 ± 34.3
Plasma triglycerides (mg/dL)	130.4 ± 81.5	125.3 ± 84.8	128.2 ± 82.9	112 ± 66.6	112.1 ± 60.2	112.1 ± 62.7	121.6 ± 75.6
***Dietary intake***							
Energy intake (kcal)	2378 ± 553	2526 ± 576	2442 ± 567	1884 ± 463	2010 ± 523	1960 ± 503^c^	2243 ± 591
Total carbohydrates (g/d)	233.3 ± 65.0	246.2 ± 64.5	238.9 ± 65.0	198.0 ± 72.7	210.2 ± 65.3	205.3 ± 68.5^c^	225.0 ± 68.4
Total protein (g/d)	86.4 ± 23.2	90.6 ± 22.0	88.2 ± 22.8	65.4 ± 16.2	69.3 ± 20.5	67.7 ± 18.9^c^	79.8 ± 23.5
Total fat (g/d)	106.3 ± 28.7	112.2 ± 29.1	108.9 ± 29.0	83.3 ± 19.4	88.7 ± 26.2	86.5 ± 23.8^*c*^	99.6 ± 29.1
n-6:n-3 ratio	5.9 ± 1.4	5.5 ± 1.2	5.7 ± 1.3	6.0 ± 1.4	5.5 ± 1.3^b^	5.7 ± 1.4	5.7 ± 1.3
Total fiber (g/d)	21.6 ± 6.2	24 ± 6.3^b^	22.6 ± 6.4	19.8 ± 5.7	22.5 ± 6.0^b^	21.4 ± 6.0	22.1 ± 6.2
Alcohol (g/d)	17.1 ± 17.8	21.1 ± 23.7	18.9 ± 20.6	8.8 ± 10.1	10.2 ± 13.1	9.7 ± 12.0^c^	15.1 ± 18.1
***Smoking status*** [*n* (%)]							
Never	88 (45)	59 (40)	147 (43)	54 (56)	90 (62)	144 (60)	291 (50)
Former	87 (45)	81 (55)	168 (49)	31 (32)	40 (28)	71 (29)^c^	239 (41)
Current	19 (10)	8 (5)	27 (8)	12 (12)	14 (10)	26 (11)	53 (9)
***Types of physical activity***							
Total MET-h/wk	59.0 ± 22.0	155.1 ± 70.7^b^	100.6 ± 68.6	64.4 ± 21.4	160.3 ± 54.0^b^	121.7 ± 64.4^c^	109.3 ± 67.6
Housework (h/wk)	2.9 ± 2.9	6.6 ± 6.0^b^	4.5 ± 4.9	7.5 ± 5.0	17.0 ± 9.4^b^	13.2 ± 9.2^c^	8.1 ± 8.2
Walking (h/wk) ^d^	4.3 ± 3.4	10.4 ± 9.7^b^	6.9 ± 7.5	4.6 ± 3.3	10.2 ± 8.4^b^	7.9 ± 7.4	7.4 ± 7.5
Gardening (h/wk) ^d^	1.7 ± 2.2	5.3 ± 6.4^b^	3.3 ± 4.9	1.1 ± 1.7	4.2 ± 5.1^b^	2.9 ± 4.4	3.1 ± 4.7
Do-it-yourself work (h/wk)	1.5 ± 1.5	4.5 ± 4.4^b^	2.8 ± 3.5	0.4 ± 1.0	1.1 ± 2.4^b^	0.8 ± 2.0^c^	2.0 ± 3.1
Stair climbing (flights/d) ^d^	5.0 ± 5.5	7.1 ± 7.8^b^	5.9 ± 6.7	3.7 ± 3.7	5.6 ± 8.0	4.8 ± 6.7	5.5 ± 6.7
Sports (h/wk)	1.8 ± 1.9	3.8 ± 4.0^b^	2.7 ± 3.2	1.6 ± 1.5	3.5 ± 4.1^b^	2.7 ± 3.5	2.7 ± 3.3
Cycling (h/wk) ^d^	1.3 ± 1.5	4.6 ± 4.4^b^	2.7 ± 3.5	0.9 ± 1.3	3.3 ± 3.7^b^	2.4 ± 3.2	2.6 ± 3.4
Total activity reported (h/d)	2.0 ± 0.7	5.1 ± 2.5^b^	3.3 ± 2.3	2.3 ± 0.8	5.6 ± 1.9^b^	4.3 ± 2.3^c^	3.7 ± 2.3
***Types of inactivity***							
Sleeping (h/d) ^d^	7.5 ± 1.3	7.6 ± 1.0	7.6 ± 1.2	7.7 ± 1.4	7.6 ± 1.6	7.6 ± 1.5	7.6 ± 1.3
Watching TV (h/d)	2.9 ± 1.4	2.8 ± 1.3	2.9 ± 1.4	3.1 ± 2.2	2.8 ± 2.5	3.0 ± 2.3	2.9 ± 1.8
Total inactivity reported (h/d)	10.4 ± 2.0	10.4 ± 1.7	10.4 ± 1.9	10.8 ± 2.8	10.4 ± 3.6	10.6 ± 3.3	10.5 ± 2.5
Overall 24-h inactivity (h/d) ^e^	22.0 ± 0.7	18.9 ± 2.5^b^	20.7 ± 2.3	21.7 ± 0.8	18.4 ± 1.9^b^	19.7 ± 2.3^c^	20.3 ± 2.3
***Volumes of adipose tissue*** ^f^							
VAT (dm^3^) ^d^	5.1 ± 2.0	4.8 ± 2.1	4.9 ± 2.1	3.0 ± 1.5	2.8 ± 1.5	2.9 ± 1.5^c^	4.1 ± 2.1
SAAT (dm^3^) ^d^	6.4 ± 2.9	5.7 ± 2.2^b^	6.1 ± 2.7	8.9 ± 4.7	7.7 ± 3.6	8.2 ± 4.1^c^	7.0 ± 3.5
STRAT (dm^3^) ^d^ ^,^ ^g^	9.2 ± 3.8	8.2 ± 3.1^b^	8.7 ± 3.5	12.5 ± 6.2	10.9 ± 4.7	11.5 ± 5.4^c^	9.9 ± 4.6

^*a*^ Data are crude arithmetic,means (± SD) or *n* (%). Differences between men and women or subjects with low total PA (MET-h/wk < 96.1) and high total PA (MET-h/wk ≥ 96.1) were assessed by using Student’s *t* test for, where necessary, transformed normalized continuous variables and a *χ* ^*2*^ test for categorical variables. *P* values are two-sided and uncorrected. The B-Y FDR threshold for statistical significance of 38 possibly dependent tests is *P* ≤ 0.012. *Abbreviations*: B-Y FDR; Benjamini-Yekutieli false discovery rate; BMI, body mass index (kg/m^2^); n-6:n-3 ratio; ratio of n-6 to n-3 fatty acids; HbA1c, glycated hemoglobin A1c; MET-h/wk, metabolic equivalent task hours per week (including housework, walking, gardening, do-it-yourself work, stair climbing, sports, cycling); PA, physical activity; SAAT, subcutaneous abdominal adipose tissue; STRAT, subcutaneous trunk adipose tissue; VAT, visceral abdominal adipose tissue.

^*b*^ Different at B-Y FDR significance level from subjects of the same gender with low PA (MET-h/wk < 96.1) (*P* ≤ 0.012).

^*c*^ Different at B-Y FDR significance level from male subjects (*P* ≤ 0.012).

^*d*^ For Student’s *t* tests, non-normally distributed variables were transformed to reach normality using the natural logarithm (BMI, C-reactive protein, HbA1c, sleep, SAAT, STRAT) or square-root (walking, cycling, sports, gardening, stair climbing, watching TV, VAT) transformation.

^*e*^ Assumed overall 24-h inactivity (h/d) was calculated as the difference between 24 hours and total PA reported (h/d).

^*f*^ Adipose tissue: 1 dm^3^ = 1 L = ~0.9 kg.

^*g*^ Subcutaneous trunk adipose tissue was measured from the humeral to the femoral heads.

### PA MET and VAT and SAAT

In multivariable analysis adjusted for BMI (model 1) (Table 2), PA MET was negatively associated with VAT, but not SAAT (*P* = 0.3). Moreover, the association with VAT was modified by sex, with a significant negative association in men, but not women. For both VAT and SAAT, the associations with PA MET were not modified by median age or median BMI.

**Table 2:** Association of metabolic equivalents of reported total physical activity with visceral and subcutaneous abdominal adipose tissue unstratified and stratified by sex, age, and BMI in white adults.^a^

		VAT (cm^3^)						SAAT (cm^3^)					
Total and strata of PA MET		Model 1			Model 2			Model 1			Model 2		
	(n)	*B*	(SE)	*P*	*B*	(SE)	*P*	*B*	(SE)	*P*	*B*	(SE)	*P*
**Total**	583	-1.4	(0.7)	0.04	-2.8	(0.9)	0.003	-0.9	(0.8)	0.3	-2.7	(1.6)	0.08
***Sex***													
M	342	-2.6	(0.9)	0.006	-3.3	(1.4)	0.02	-0.9	(0.9)	0.3	-1.7	(1.6)	0.3
F	241	-0.2	(0.8)	0.8	-2.1	(1.1)	0.07	-0.7	(1.4)	0.6	-5.7	(2.5)	0.05
*P*interaction				0.011			0.7			0.7			0.4
***Age (y)***													
<62.0	293	-0.7	(0.8)	0.4	-1.4	(1.1)	0.2	-0.2	(1.1)	0.8	-0.3	(2.2)	0.98
≥62.0	293	-1.8	(1.2)	0.1	-3.7	(1.5)	0.016	-2.7	(1.2)	0.02	-5.7	(2.2)	0.008
*P*interaction				0.5			0.5			0.1			0.2
***BMI (kg/m*** ^***2***^ ***)***													
<26.7	294	-2.2	(0.9)	0.009	-2.3	(0.9)	0.009	-2.0	(1.4)	0.1	-2.0	(1.4)	0.1
≥26.7	289	-2.8	(1.3)	0.03	-2.3	(1.2)	0.06	-2.7	(1.9)	0.1	-2.4	(1.8)	0.2
*P*interaction				0.9			0.9			0.7			0.7

^*a*^ Data (*n* = 583 subjects) are unstandardized regression coefficients (*B*) of robust multiple linear regression models adjusted for age (except age strata), sex (except sex strata), smoking status (never, former, and current smoker) and either BMI (except BMI strata) (model 1) or height (model 2). Regression coefficients (*B*) indicate the impact of one unit change in metabolic equivalents of reported PA MET on VAT or SAAT (cm^3^; 1 cm^3^ = 1 mL = ~0.9 g), i.e. after adjusting for covariates, an increase in PA MET by one unit was associated with a change of *B* units (cm^3^) in AAT. *P* values are two-sided and uncorrected. *Abbreviations*: BMI, body mass index (kg/m^2^); F, females; M, males; PA MET, metabolic equivalent hours per week of total physical activity (including housework, walking, gardening, do-it-yourself work, stair climbing, sports, cycling); SAAT, subcutaneous abdominal adipose tissue; VAT, visceral abdominal adipose tissue.

In the corresponding multivariable analysis adjusted for height (final model 2, Table 2), similar to BMI adjustment, PA MET was negatively associated with VAT, but not SAAT (*P* = 0.08). Moreover, the associations between PA MET and both VAT and SAAT were not modified by sex, median age, or median BMI. In the respective sex, age, or BMI strata, however, the association with VAT was significant in men, subjects aged ≥62.0 y, and subjects with BMI < 26.7 kg/m^2^ only, whereas the association with SAAT was significant in women and subjects aged ≥62.0 y only.

**Fig 1:**
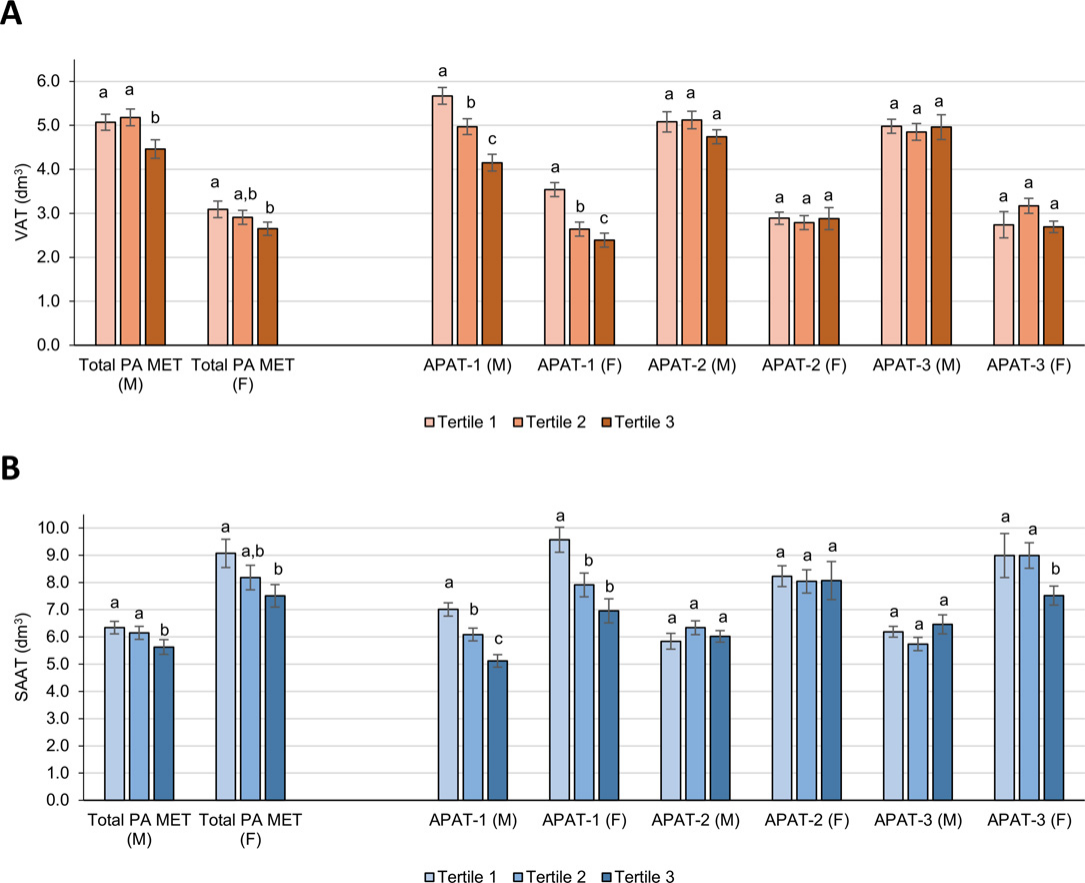
Total volumes of VAT and SAAT for tertiles of total physical activity (MET-h/wk) and for PCA-derived activity patterns by sex (M/ F) in Northern German adults. Data (n = 583 subjects) are LSM (± SE) of total volumes of **(1)** VAT or **(2)** SAAT from generalized linear models adjusted for age, total energy intake (kcal/d), smoking status (never, former, and current smoker), and height (final model 2) by tertile category and sex of PA MET and PCA-derived activity patterns (for exact data see also S2 Table). Multiple comparisons assessing statistical differences in LSM between tertiles were corrected by using the Tukey-Kramer procedure. Mean values without sharing a common superscript letter (a-c) were statistically different at *P* < 0.05. APAT 1–3; activity pattern 1 to 3 derived by PCA; B-Y FDR, Benjamini-Yekutieli false discovery rate; F, females; LSM, least-square mean; M, males; PA MET, metabolic equivalent hours per week of total physical activity (including housework, walking, gardening, do-it-yourself work, stair climbing, sports, cycling); PCA, principal components analysis; SAAT, subcutaneous abdominal adipose tissue; VAT, visceral abdominal adipose tissue.

Stratified by sex and tertile category of PA MET (S2 Table and Fig 1), in multivariable analysis adjusted for height (final model 2), there was a non B-Y FDR significant inverse linear trend (*P*_trend_ < 0.05) for the association between PA MET and VAT and SAAT in both men and women. However, compared with subjects in the lowest tertile (PA MET < 74.1), volumes of VAT or SAAT, respectively, were only significantly lower among men (-0.61 dm^3^ [-12.0%] or -0.71 dm^3^ [-11.2%]) and women (-0.44 dm^3^ [-14.2%] or -1.56 dm^3^ [-17.2%]) in the highest tertile (PA MET > 121.9), but not among subjects in the second tertile.

### APAT derived by PCA

**Fig 2:**
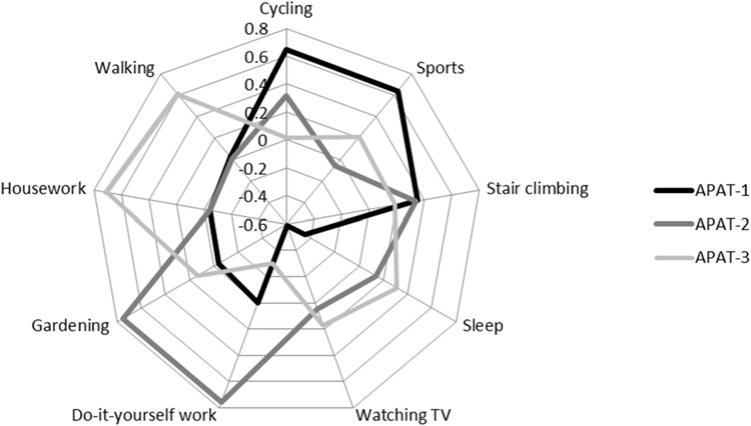
PCA-derived activity patterns in Northern German adults (*n* = 583 subjects). Identified by PCA, on the circular plot the first three principal components that met the Scree test and eigenvalue >1.0 criterion are shown by different colored lines (referred to as APAT pattern 1 to 3). For each of the 7 physical activity and 2 inactivity items, the component loadings of the individual 3 patterns are indicated on the circular axis (component loading scores ranging between -0.6 and +0.7). Each component represents an independent activity pattern including all activity items that yielded a component loading ≥0.5 for this pattern. Activity items that did not obtain a component loading ≥0.5 for any of the principal components were assigned to the pattern the component loading of which was highest. Activity items with similarly high loadings for two patterns were regarded as representative of both patterns. The variation (%) in activity variables explained by APAT 1–3 was 16.8, 15.4, and 13.3 (overall 45.5), respectively. PCA, principal components analysis.

Three major principal components representative of independent APAT (APAT-1 to APAT-3) were identified by PCA (Fig 2). With regard to activities loading high on a specific pattern, APAT-1 included cycling and sports; APAT-2 encompassed DIY work and gardening; and APAT-3 comprised housework, walking, sleep, and watching TV. Stair climbing loaded similarly high on both APAT-1 and APAT-2 and, therefore, was regarded as representative of both patterns. The variation (%) in activity variables explained by APAT 1–3 was 16.8, 15.4, and 13.3 (overall 45.5), respectively.

### Types of reported PA, IA, or APAT and VAT and SAAT

In multivariable analysis adjusted for BMI (model 1) (Table 3), considering B-Y FDR significance (*P* ≤ 0.015), VAT was negatively associated with time spent cycling and APAT-1, and positively with time spent watching TV and total IA reported. The associations with APAT-1, watching TV, and total IA reported were modified by sex, with APAT-1 revealing a stronger negative association in men than women, and watching TV (*P* = 0.02) and total IA reported (*P* = 0.07) showing a stronger non B-Y FDR significant positive association in men than women. Similarly, the association between stair climbing and VAT was modified by sex, with a non B-Y FDR significant negative association in men (*P* = 0.04), but not women. For SAAT, only effect modification by sex for DIY work was revealed with a non B-Y FDR significant negative association in men (*P* = 0.02), but not women.

**Table 3:** Association of reported individual types of physical activity and inactivity or PCA-derived activity patterns with visceral and subcutaneous abdominal adipose tissue unstratified and stratified by sex in white adults.^a^

		VAT (cm^3^)						SAAT (cm^3^)					
		Model 1			Model 2			Model 1			Model 2		
Type or pattern of activity	(n)	*B*	(SE)	*P*	*B*	(SE)	*P*	*B*	(SE)	*P*	*B*	(SE)	*P*
**Physical activities**													
*Housework (h/wk)*													
Total	583	7.8	(7.2)	0.3	4.8	(10.0)	0.6	-5.1	(8.5)	0.6	5.0	(16.6)	0.8
*Sex*													
*M*	342	15.4	(14.5)	0.3	45.8	(21.9)	0.04	12.5	(13.9)	0.4	44.4	(23.6)	0.06
*F*	241	0.3	(6.3)	0.96	-4.7	(9.1)	0.6	-6.3	(11.1)	0.6	-27.2	(27.2)	0.3
*P*_interaction_				0.2			0.02			0.4			0.1
*DIY work (h/wk)*													
Total	583	-35.5	(17.0)	0.04	14.4	(23.6)	0.5	-26.3	(20.1)	0.2	65.5	(39.2)	0.1
*Sex*													
M	342	-72.5	(20.7)	**0.0005** ^b^	-2.8	(32.0)	0.9	-48.5	(20.1)	0.02	30.0	(34.4)	0.4
F	241	39.6	(27.5)	0.1	114.4	(39.9)	**0.004**	48.5	(49.2)	0.3	328.5	(118.1)	**0.005**
*P*_interaction_				0.2			0.05			**0.011**			**0.003**
*Gardening (h/wk)*													
Total	583	-3.4	(10.8)	0.8	7.6	(14.9)	0.6	-8.4	(12.7)	0.5	33.6	(24.7)	0.2
*Sex*													
M	342	-18.6	(14.9)	0.2	9.5	(22.6)	0.7	-6.8	(14.3)	0.6	25.0	(24.2)	0.3
F	241	15.2	(12.7)	0.2	16.1	(18.5)	0.4	-13.1	(22.7)	0.6	39.5	(55.7)	0.5
*P*_interaction_				0.5			0.8			0.99			0.8
*Walking (h/wk)*													
Total	583	-0.7	(6.7)	0.9	-14.8	(9.3)	0.1	-2.0	(7.9)	0.8	-31.7	(15.3)	0.04
*Sex*													
M	342	-7.5	(9.5)	0.4	-13.7	(14.6)	0.3	-1.0	(9.2)	0.9	-9.7	(15.6)	0.5
F	241	-0.3	(7.6)	0.97	-18.4	(10.8)	0.09	-2.6	(13.5)	0.9	-64.0	(32.4)	0.05
*P*_interaction_				0.1			0.6			0.4			0.1
*Stair climbing (stairs/d)*													
Total	583	-11.6	(7.5)	0.1	-30.9	(10.4)	**0.003**	7.7	(8.9)	0.4	-27.4	(17.3)	0.1
*Sex*													
M	342	-22.3	(10.7)	0.04	-42.0	(16.3)	**0.010**	0.6	(10.4)	0.95	-18.9	(17.7)	0.3
F	241	1.6	(8.4)	0.8	-16.1	(12.1)	0.2	18.3	(14.8)	0.2	-51.0	(36.1)	0.2
*P*_interaction_				**0.004**			0.3			0.6			0.6
*Cycling (h/wk)*													
Total	583	-42.0	(14.8)	**0.004**	-78.1	(20.2)	**0.0001**	-26.8	(17.4)	0.1	-88.8	(33.7)	**0.008**
*Sex*													
M	342	-40.0	(20.7)	0.05	-106.9	(30.7)	**0.0005**	-28.4	(19.9)	0.2	-97.3	(33.1)	**0.003**
F	241	-29.7	(16.9)	0.08	-46.4	(24.5)	0.06	-39.2	(30.1)	0.2	-65.2	(74.3)	0.4
*P*_interaction_				0.2			0.2			0.6			0.4
*Sports (h/wk)*													
Total	583	-34.5	(14.9)	0.02	-63.5	(20.7)	**0.002**	-23.0	(17.7)	0.2	-65.8	(34.5)	0.06
*Sex*													
M	342	-45.1	(22.2)	0.04	-92.3	(33.8)	**0.006**	-24.8	(21.5)	0.2	-80.7	(36.5)	0.03
F	241	-15.3	(15.8)	0.3	-38.2	(22.9)	0.1	-24.5	(28.0)	0.4	-78.8	(68.7)	0.3
*P*_interaction_				0.08			0.2			0.7			0.8
**Inactivities**													
*Sleeping (h/d)*													
Total	583	43.0	(36.8)	0.2	93.1	(51.1)	0.07	92.3	(43.4)	0.03	155.1	(84.9)	0.07
*Sex*													
M	342	5.4	(59.1)	0.9	79.3	(90.1)	0.4	32.4	(56.7)	0.6	59.3	(96.8)	0.5
F	241	57.7	(36.3)	0.1	88.0	(52.7)	0.2	91.4	(64.5)	0.2	277.1	(157.5)	0.08
*P*_interaction_				0.9			0.9			0.3			0.2
*Watching TV (h/d)*													
Total	583	71.3	(28.1)	**0.011**	224.9	(36.9)	**<0.0001**	10.3	(33.5)	0.8	354.9	(62.1)	**<0.0001**
Sex													
M	342	123.9	(53.9)	0.02	462.8	(74.8)	**<0.0001**	56.2	(52.3)	0.3	370.4	(84.1)	**<0.0001**
F	241	50.0	(24.7)	0.04	134.5	(34.6)	**0.0001**	-23.4	(44.4)	0.6	330.9	(103.9)	**0.002**
*P*_interaction_				**<0.0001**			**<0.0001**			0.6			0.97
**Activity patterns**													
*APAT-1*													
Total	583	-287.4	(50.9)	**<0.0001**	-552.9	(67.5)	**<0.0001**	-132.9	(62.0)	0.03	-670.2	(114.6)	**<0.0001**
*Sex*													
M	342	-276.7	(76.6)	**0.0003**	-712.7	(108.2)	**<0.0001**	-124.5	(75.4)	0.1	-600.1	(119.9)	**<0.0001**
F	241	-242.2	(53.5)	**<0.0001**	-396.2	(76.4)	**<0.0001**	-157.6	(98.5)	0.1	-776.0	(231.7)	**0.0008**
*P*_interaction_				**0.001**			0.03			0.6			0.4
*APAT-2*													
Total	583	-82.7	(53.4)	0.1	-54.0	(74.5)	0.5	8.9	(63.1)	0.9	168.4	(123.3)	0.2
*Sex*													
M	342	-224.3	(79.4)	**0.005**	-142.5	(121.6)	0.2	-89.7	(76.6)	0.2	29.6	(130.8)	0.8
F	241	7.8	(56.6)	0.9	66.4	(82.7)	0.4	73.3	(101.0)	0.5	398.2	(245.8)	0.1
*P*_interaction_				0.04			0.2			0.04			0.1
*APAT-3*													
Total	583	-29.6	(57.8)	0.6	-108.7	(80.1)	0.2	52.4	(68.2)	0.4	-22.3	(133.2)	0.9
*Sex*													
M	342	-103.9	(83.6)	0.2	-102.2	(128.8)	0.4	73.2	(80.7)	0.4	-2.9	(138.2)	0.98
F	241	41.9	(63.4)	0.5	-38.0	(92.5)	0.7	-7.3	(113.3)	0.95	-166.8	(277.2)	0.5
*P*_interaction_				0.08			0.7			0.3			0.96
**Total inactivity reported (h/d)**													
Total	583	50.1	(20.0)	**0.010**	148.0	(26.8)	**<0.0001**	30.1	(23.9)	0.2	242.4	(44.9)	**<0.0001**
*Sex*													
M	342	71.7	(39.7)	0.07	290.6	(56.9)	**<0.0001**	46.5	(38.4)	0.2	244.7	(63.0)	**0.0001**
F	241	36.8	(17.3)	0.03	80.2	(24.5)	**0.001**	7.6	(31.0)	0.8	227.6	(73.0)	**0.002**
*P*_interaction_				**0.0001**			**0.0002**			0.8			0.7
**Overall 24-h inactivity (h/d)** ^c^													
Total	583	25.2	(22.1)	0.3	50.5	(30.7)	0.1	35.2	(26.0)	0.2	55.0	(50.8)	0.3
*Sex*													
M	342	58.8	(31.0)	0.06	45.2	(47.8)	0.3	25.3	(30.0)	0.4	14.8	(51.2)	0.8
F	241	3.8	(25.0)	0.9	51.9	(36.1)	0.2	37.9	(44.3)	0.4	157.8	(108.0)	0.1
*P*_interaction_				0.2			0.7			0.5			0.3

^*a*^ Data (*n* = 583 subjects) are unstandardized regression coefficients (*B*) of robust multiple linear regression models adjusted for age, sex (except sex strata), smoking status (never, former, and current smoker) and either BMI (kg/m^2^) (model 1) or height (model 2). Regression coefficients (*B*) indicate the impact of one unit change in activity variables (h/wk, stairs/d, h/d, or PCA score) on VAT or SAAT (cm^3^; 1 cm^3^ = 1 mL = ~0.9 g), i.e. after adjusting for covariates, an increase in a type or pattern of physical activity by one unit was associated with a change of *B* units (cm^3^) in AAT. *P* values are two-sided and uncorrected. The B-Y FDR threshold *P* value for 14 possibly dependent tests is 0.015. Bolded *P* values indicate a statistical significance at the B-Y FDR level. *Abbreviations*: APAT 1–3; activity patterns 1 to 3 derived by PCA; BMI, body mass index (kg/m^2^); B-Y FDR, Benjamini-Yekutieli false discovery rate; DIY, do-it-yourself; F, females; M, males; PCA, principal components analysis; SAAT, subcutaneous abdominal adipose tissue; VAT, visceral abdominal adipose tissue.

^*b*^ All such bolded values: Statistically significant at B-Y FDR level (*P* ≤ 0.015).

^*c*^ Assumed overall 24-h inactivity (h/d) was calculated as the difference between 24 hours and total physical activity reported (h/d).

In the corresponding multivariable analysis adjusted for height instead of BMI (final model 2, Table 3), both VAT and SAAT were negatively associated with time spent cycling and APAT-1, and positively with time spent watching TV and total IA reported. VAT was additionally negatively associated with time spent stair climbing and time spent on sports. The associations between time spent watching TV or total IA reported and VAT were modified by sex, though, with a much stronger positive association in men than women. Moreover, the association between DIY work and SAAT was modified by sex with a positive association in women, but not men.

### Tertiles of reported IA, PA, or APAT and VAT and SAAT

**Fig 3:**
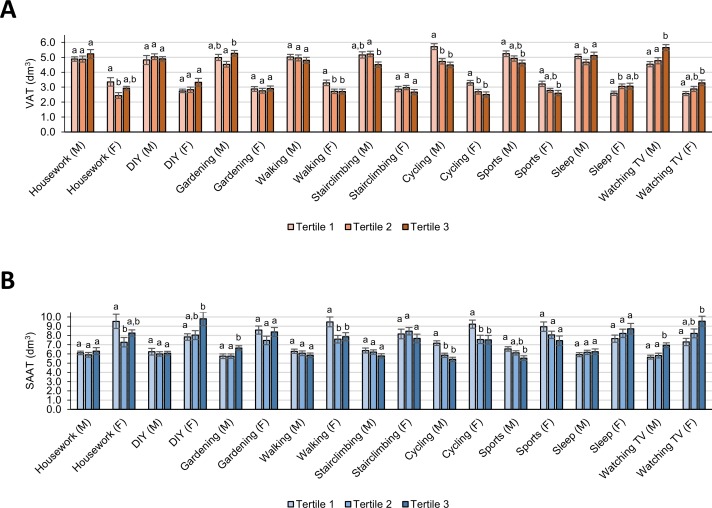
Total volumes of VAT and SAAT for tertiles of physical activities and inactivities by sex (M/F) in Northern German adults. Data (n = 583 subjects) are LSM (± SE) of total volumes of **(1)** VAT or **(2)** SAAT from generalized linear models adjusted for age, total energy intake (kcal/d), smoking status (never, former, and current smoker), and height (final model 2) by tertile category and sex of individual physical activity variables (for exact data see also S2 Table). Multiple comparisons assessing statistical differences in LSM between tertiles were corrected by using the Tukey-Kramer procedure. Mean values without sharing a common superscript letter (a-b) were statistically different at *P* < 0.05. DIY, do-it-yourself; F, females; LSM, least-square mean; M, males; SAAT, subcutaneous abdominal adipose tissue; VAT, visceral abdominal adipose tissue.

In multivariable analysis adjusted for height (final model 2), stratified by sex and tertile category of individual activities (S2 Table and Fig 3) and considering B-Y FDR significance (P ≤ 0.016), there was a quadratic trend (*P*_trend_ ≤ 0.002) between time spent on *housework* and VAT and SAAT among women, but not men. Compared to women spending <3 h/wk on housework, women had lower VAT and SAAT when 3–9 h/wk, but not when >9 h/wk, were spent on housework. As against subjects spending no time on *DIY work*, women, but not men, had higher SAAT when >1.5 h/wk, but not when ≤1.5 h/wk were spent on DIY work. For *gardening*, there was a linear trend for the association with SAAT among men (*P*_trend_ = 0.001), but not women. Compared with men spending <0.4 h/wk gardening, men had higher SAAT when >2.8 h/wk, but not when 0.4–2.8 h/wk, were spent gardening. As against subjects spending <3.0 h/wk *walking*, women, but not men, had lower VAT and SAAT when ≥3.0 h/wk were spent walking. Regarding *stair climbing*, there was a linear trend for the association with VAT in men (*P*_trend_ = 0.005), but not women. Compared to men climbing <1.0 stairs/d, men who climbed >4 stairs/d, but not those climbing 1–4 stairs/d, had lower VAT. Among both men and women, there was a linear trend for the association of *cycling* with VAT, and for men also with SAAT (*P*_trend_ ≤ 0.004). Compared with subjects spending <0.4 h/wk cycling, men and women had lower VAT or SAAT when at least ≥0.4 h/wk were spent cycling. Similarly, there was a linear trend of lower VAT with increasing hours spent on *sports* among men and women, and for men also of lower SAAT (*P*_trend_ ≤ 0.013). As against subjects spending <0.9 h/wk on sports, men and women spending >3.0 h/wk, but not those spending 0.9–3.0 h/wk, on sports had lower VAT, and males also lower SAAT. Compared with subjects spending <7.3 h/d *sleeping*, men had lower VAT when 7.3–8.2 h/d, but not when >8.2 h/d were spent, whereas women had higher VAT when ≥7.3 h/d were spent sleeping. Among both men and women, there was a linear trend (*P*_trend_ ≤ 0.007) for the association between *watching TV* and VAT and SAAT. As against subjects spending <2.0 h/d watching TV, men and women had higher VAT or SAAT when >3.0 h/d, but not when 2.0–3.0 h/d, were spent watching TV.

For both men and women, there was a highly significant linear trend (*P*_trend_ ≤ 0.0005) of lower VAT and SAAT with increasing scores of *APAT-1* (S2 Table and Fig 1), which loaded high on VPA (Fig 2). Regarding the midpoint between the VPA values (h/wk) of two respective APAT-1 tertiles (S1 Table) as the boundary value for VPA between these two tertiles, compared to subjects in the lowest tertile (<4.0 h/wk VPA), men and women in the second tertile (4.0–6.8 h/wk VPA) as well as men (-1.52 dm^3^ [-26.8%] or -1.89 dm^3^ [-27.0%]) and women (-1.15 dm^3^ [-32.5%] or -2.61 dm^3^ [-27.3%]) in the third tertile (>6.8 h/wk VPA) had lower VAT or SAAT, respectively. However, in both men and women, there was no association of *APAT-2* or *APAT-3* loading high on MPA or MPA and IA, respectively (Fig 2 and S1 Table), with either VAT or SAAT except for APAT-3 and SAAT in women. Compared to tertiles of total PA MET that quantitatively summarizes different types of PA in terms of energy expenditure, of the APAT that qualitatively group individual PA according to related PA behaviors, particularly tertiles of APAT-1 (explaining most of the variation in PA behaviors) showed a similar pattern of association with both VAT or SAAT (S2 Table and Fig 1). However, for both men and women, respectively, the tertiles of APAT-1 captured more of the variance in VAT (26.8% and 32.5%) and SAAT (27.0% and 27.3%) than the tertiles of PA MET with regard to VAT (14.2% and 14.2%) and SAAT (17.7% and 17.2%).

Overall, about two third of the 17 activity item or pattern variables investigated were significantly related to VAT or SAAT in BMI- or height-adjusted analysis (Table 4). Most results from models including either an individual PA or IA item or an APAT variable containing this item obtained similar results, i.e. individual PA or IA loading high on a APAT found to be significantly associated with VAT or SAAT were also related to the respective AAT when the respective PA or IA was analyzed individually.

**Table 4:** Overview of individual physical activities, inactivities, and overall activity patterns significantly associated with visceral or subcutaneous abdominal adipose tissue Northern German adults using multivariable BMI- or height-adjusted analysis.^a^

	VAT (dm^3^)				SAAT (dm^3^)			
	Activity item	Association	Relevance of sex	Threshold / range	Activity item	Association	Relevance of sex	Threshold / range
**Continuous PA, IA, and APAT variables**								
(linear regression)								
**Model 1**								
(BMI-adjusted)								
Total PA MET and IA	PA MET	(—)	(M)/I_M_	n/a				
	Total IA	(+)	I_(F)_	n/a				
Individual PA and IA	DIY	(—)	M	n/a	DIY	(—)	I_(M)_	
	Stair climbing	(—)	I_(M)_	n/a				
	Cycling	(—)						
	Watching TV	(+)	I_(M/F)_	n/a				
Activity patterns	APAT-1	(—)	I_M/F_	n/a				
	APAT-2	(—)	M	n/a				
**Model 2**								
(height-adjusted)								
Total PA MET and IA	PA MET	(—)	(M)	n/a	PA MET	(—)	F	n/a
	Total IA	(+)	I_M/F_	n/a	Total IA	(+)		n/a
Individual PA and IA	DIY	(+)	F	n/a	DIY	(+)	I_F_	n/a
	Stair climbing	(—)	(M)	n/a				
	Cycling	(—)	(M)	n/a	Cycling	(—)	(M)	n/a
	Sports	(—)	(M)	n/a				
	Watching TV	(+)	I_M/F_	n/a	Watching TV	(+)		n/a
Activity patterns	APAT-1	(—)		n/a	APAT-1	(—)		n/a
**PA, IA, and APAT tertiles**								
(ANCOVA)								
**Model 2**								
(height-adjusted)								
Total PA MET	PA MET	(—)		>122 MET-h/wk ~ lower VAT	PA MET	(—)		>122 MET-h/wk ~ lower SAAT
Individual PA or IA	Housework	U-shaped	F	3.0–9.0 h/wk ~ lower VAT	Housework	U-shaped	F	3.0–9.0 h/wk ~ lower SAAT
					DIY	(+)	F	>1.5 h/wk ~ higher SAAT
					Gardening	(+)	M	>2.8 h/wk ~ higher SAAT
	Walking	(—)	F	>3.0 h/wk ~ lower VAT	Walking	(—)	F	>3.0 h/wk ~ lower SAAT
	Stair climbing	(—)	M	>4 stairs/d ~ lower VAT				
	Cycling	(—)		>0.4 h/wk ~ lower VAT	Cycling	(—)		>0.4 h/wk ~ lower SAAT
	Sports	(—)		>3 h/wk ~ lower VAT	Sports	(—)	M	>3.0 h/wk ~ lower SAAT
	Sleep	U-shaped	M	7.3–8.2 h/d ~ lower VAT				
		(+)	F	<7.3 h/d ~ lower VAT				
	Watching TV	(+)		>3.0 h/d ~ higher VAT	Watching TV	(+)		>3.0 h/d ~ higher SAAT
Activity patterns	APAT-1	(—)		>4.0 h/wk VPA ~ lower VAT	APAT-1	(—)	-	>4.0 h/wk VPA ~ lower SAAT

^*a*^ Multivariable robust or generalized linear regression models (*n* = 583 subjects) were adjusted for age, sex (except sex strata), total energy intake, smoking status (never, former, and current smoker), and either body mass index (kg/m^2^) (model 1) or height (model 2). Activity patterns were derived by PCA. Of the activity items and patterns explored, only significant results that reached the B-Y FDR threshold of statistical significance (*P* ≤ 0.015 for 14 individual activity items and patterns) are presented. (+), positive association; (—), inverse association; U-shaped, quadratic U-shaped association. “M” and “F”, respectively, indicate that the association was significant among men or women only; “(M)” and “(F)”, respectively, indicate that the association was significant in the total population, however, when stratified by sex remained significant among men or women only; “I_M_” and “I_F_” indicate a B-Y FDR significant interaction effect by sex with a B-Y FDR significant positive or negative association in men or women, respectively; “I_(M)_” and “I_(F)_” indicate a B-Y FDR significant interaction effect by sex with a non B-Y FDR significant positive or negative association (*P* ≤ 0.05) in men or women, respectively. *Abbreviations*: ANCOVA, analysis of covariance; APAT, activity pattern; APAT-1 to APAT-3, activity pattern 1 to 3 derived by PCA; B-Y FDR, Benjamini-Yekutieli false discovery rate; DIY, do-it-yourself work; F, females; I, interaction effect; IA; inactivity; M, males; PA, physical activity; PA MET, metabolic equivalent hours per week of total physical activity (including housework, walking, gardening, DIY, stair climbing, sports, cycling); PCA, principal components analysis; SAAT, subcutaneous abdominal adipose tissue; VAT, visceral abdominal adipose tissue; VPA, vigorous PA.

## Discussion

The present study is the first to report associations of APAT and individual types of PA and IA with total volumes of VAT and SAAT by sex for a general population sample. Whereas habitual VPA were negatively associated with VAT and SAAT in both white men and women, associations for habitual MPA and IA were usually depended on sex and/or a specific threshold or range for a specific activity volume. Moreover, habitual APAT including ≥4.0–6.8 h/wk VPA, but not APAT rich in MPA only, were associated with lower total volumes of VAT and SAAT.

### PA intensity and VAT and SAAT

In line with our study, a number of intervention studies have reported that VPA is able to reduce VAT and SAAT in both men and women [21–25]. Few observational studies also found a negative association of VPA [17, 27] or moderate-to-vigorous PA (MVPA) [17, 18] with VAT in adults, and even of sporadic MVPA [18, 32], i.e. bouts lasting <10 min, similar to the negative association between stair climbing and VAT in men in our study. As with our study showing a linear association between VPA and VAT, a meta-analysis of intervention studies found a dose-response relation between aerobic exercise and VAT reduction in healthy obese subjects [53]. With regard to SAAT, however, results of associations with higher-intensity PA are conflicting [17]. Dependent on ethnicity, a negative association [17], but also no association [17, 18] of VPA or MVPA with SAAT have been reported. For MPA and light-intensity PA, associations with VAT and SAAT are also not clear. We found that associations for MPA were dependent on sex. Similarly, whereas two intervention studies in men [37] or both sexes [54] did not find a reduction in VAT and SAAT due to MPA, two cross-sectional studies in women [55] or both sexes [17] found a negative association between MPA and VAT [17, 55], but not SAAT [17]. Likewise, in line with the negative association of walking with VAT and SAAT in women, but not men, in our study, an intervention study including ~73% women reported that light-intensity walking was capable of reducing VAT [38].

### Sex and other factors in the association between PA and AAT

In our study, we revealed a stronger negative association of PA MET or higher-intensity PA with VAT in men, whereas with SAAT in women. Similarly, overall PA was stronger negatively associated with waist circumference in men than women in a prospective cohort study [7]. Biologically, a stronger association between MVPA or VPA and VAT in men than women may be explained by an interplay of 1) the presence of higher VAT in men than women [1, 2, 16]; 2) the observation that men usually perform the same MVPA [56] and probably also VPA at higher intensities than do women; and 3) the physiology of visceral adipocytes that are more responsive to exercise-induced lipolytic catecholamines than subcutaneous adipocytes [4, 16]. As with our results, given that men usually carry greater VAT than women, whereas women have more SAAT than men [1, 2, 16], associations between PA and percentage values of AAT may not be directly comparable between men and women. In addition, given that there is a possible sex difference in the actual intensity of PA defined as moderate or vigorous, analyses on PA and health-related outcomes should always be performed separately for men and women. Apart from dependence on sex, inconsistencies in associations between MPA and VAT or SAAT may result from a specific threshold for an activity volume necessary to impact AAT as we found in our study. Such a threshold has also been reported for associations between MPA [38, 53] or PA MET [16] and VAT and/or SAAT. Generally, there seems to be an interplay between activity intensity and activity volume for the impact of PA on AAT [37, 57]. Ethnicity has also been found to influence the relation between PA and VAT or SAAT. Similar to our study, Europeans [17] and white and black women [30], but not Chinese and South Asians [17], have shown a consistent negative linear relationship [30].

When adjusting for BMI to investigate whether associations were independent of overall obesity, in our study the associations were usually weaker compared to height-adjustment, but especially for VAT in part still significant suggesting that PA may be associated with VAT independent of total body fat. Similarly, adjusted for total fat mass, associations between total PA [35] or VPA [58] and AAT were substantially weakened, but as opposed to SAAT still significant for VAT [35].

### IA and VAT and SAAT

We did not find any association of assumed overall 24-h IA and APAT rich in IA with both VAT and SAAT. Similarly, in a cross-sectional study, sedentary behavior was not associated with VAT or SAAT [18]. However, it has to be taken into account that our 24-h IA variable assumed that time that was not reported was spent at rest. Because of the high average age of the study population (61.0 ± 11.9 y), it is likely that the majority of the time that was not reported was indeed spent at rest or otherwise on light-intensity or MPA. Still, it cannot be ruled out that some subjects spent a substantial part of the time they did not report also on VPA, e.g. if their leisure or working time included heavy manual activities that were not covered by the questionnaire. Equally to our study showing a marked positive association of reported IA or spending ≥3 h/d watching TV with both VAT and SAAT, other cross-sectional studies reported that subjects with physical disabilities had higher VAT and SAAT [16], and subjects spending >3 h/d watching TV had higher waist circumference [59]. The latter observation was reported to be not related to a reduction in overall leisure-time PA and, therefore, may partially explained by food and beverage consumption during TV viewing [59]. Similar to other studies [60, 61], our study suggests an optimal range for sleep duration of around 8 h/d for men and possibly 7 h/d for women to be associated with lower VAT. This implies that having both inadequate (e.g. due to chronic stress) and too long sleep duration may result in accumulation of VAT compared to adequate sleep duration of 7–8 h/d.

### APAT and VAT and SAAT

Given that during moderate but not greater weight loss, preferential loss of VAT relative to SAAT has been reported [22] and accumulation of VAT is associated with greater metabolic disturbances than an increase in SAAT [1, 4], habitual long-term activity patterns resulting in stable, lower weight may be more effective to reduce VAT and improve metabolic health than short-term exercise interventions that result in rapid and more severe loss of weight. Therefore, our finding that an activity pattern including ≥4.0–6.8 h/wk VPA was associated with significantly lower AAT in white men and women may be interpreted as a recommendation for habitual PA being capable of achieving stable, low levels of VAT and SAAT. Likewise, for MPA, weekly expenditure of 1601–2283 kcal (i.e. about 6.4–9.1 h/wk MPA assuming MPA with expenditure of 250 kcal/h) has been identified to prevent excess VAT [55]. Regarding the current WHO recommendations for adults claiming that 2.5–5.0 h/wk MPA or 1.25–2.5 h/wk VPA should be spent to achieve or maintain good health, these recommendations would not be sufficient to prevent excess VAT or ensure a stable, low level of VAT.

### Strengths and limitations

Our study combines the strengths of analyzing APAT and different types of PA and IA by sex as well as the accurate assessment of total volumes of AAT. Still, the study has several limitations. First, the cross-sectional design that is prone to reverse causation and does not allow for cause-effect inference. Second, the use of a questionnaire, which is susceptible to measurement error and limited in accuracy, to estimate self-reported activities. Self-reported answers may suffer from a number of biases and articfacts including over- or understatement due to embarrassement to give true answers or recall bias resulting in over- or underestimation because of inaccurate or incomplete recollections by study participants regarding events from the past. Moreover, for individual self-reported PA that are assigned a specific MET there might be differences in the actual intensity of activities between men and women. Third, our analysis only included common activities and did not differentiate subtypes of activities. Moreover, the rather small sample sizes in stratified analysis may have attenuated statistical significance. Lastly, prospective large-scale studies are needed to confirm our results.

## Conclusions

Our findings suggest a linear negative association of habitual VPA especially with VAT, but also SAAT, in white men and women. Associations for habitual MPA and IA, however, may depend on sex and/or a threshold or range for a specific activity volume. In white populations, habitual APAT including ≥4.0–6.8 h/wk VPA, but not APAT rich in MPA only, may in the longer term help prevent excess accumulation of VAT and SAAT, which, however, remains to be confirmed in prospective studies. Given that excess VAT is a major cardiometabolic risk factor, current WHO and other national guidelines do not recommend sufficient VPA to have a meaningful beneficial impact on VAT.

## Supporting Information

S1 Table Characteristics of white adult study subjects by tertiles of exploratory activity patterns derived by principal components analysis. (PDF)

S2 Table Total volumes of visceral and subcutaneous abdominal adipose tissue for tertiles of PA MET, individual types of physical activity or inactivity, and activity patterns by sex in Northern German adults. (PDF)
