# Supplementary material for: Association of Habitual Patterns and Types of Physical Activity and Inactivity with MRI-Determined Total Volumes of Visceral and Subcutaneous Abdominal Adipose Tissue in a General White Population
Source: PLoS One. 2015 Nov 30;10(11):e0143925. doi: 10.1371/journal.pone.0143925 (PMC4664408; doi:10.1371/journal.pone.0143925)
Supplement: S1 Table — (PDF) [file pone.0143925.s001.pdf]

Supporting Information

"Association of habitual patterns and types of physical activity and inactivity with MRI-determined total volumes of visceral and subcutaneous abdominal adipose tissue in a general white population" *PLOS ONE*

Karina Fischer, Daniela Moewes, Hans-Peter Müller, Gunnar Jacobs, Jan Kassubek, Wolfgang Lieb, Ute Nöthlings

Corresponding author: Karina Fischer, Department of Geriatrics, University Hospital Zurich & Centre on Aging and Mobility, University of Zurich, Zurich, Switzerland. E-mail: karina.fischer@uzh.ch

S1 Table. Characteristics of white adult study subjects by tertiles of exploratory activity patterns derived by principal components analysis.[a]

	Exploratory activity patterns derived by PCA									
	APAT-1 rich in vigorous PA (METs 6.0-6.8)			APAT-2 rich in moderate PA (METs 3.8-4.5)			APAT-3 rich in moderate PA (METs 3.5) and IA (METs 0.95-1.3)			Total population
	Tertile 1	Tertile 2	Tertile 3	Tertile 1	Tertile 2	Tertile 3	Tertile 1	Tertile 2	Tertile 3	
Subjects [*n* (%)]	194 (33.3)	195 (33.4)	194 (33.3)	194 (33.3)	195 (33.4)	194 (33.3)	194 (33.3)	195 (33.4)	194 (33.3)	583
Age (y)	64.9 ± 10.8	60.7 ± 12.4	57.4 ± 11.2	60.6 ± 13.4	59.9 ± 11.4	62.4 ± 10.7	59.3 ± 12.2	60 ± 11.8	63.6 ± 11.4	61.0 ± 11.9
Weight (kg)	83.3 ± 16.8	80.4 ± 16.1	77.9 ± 13.9	76.9 ± 16.1	80.8 ± 16.9	83.9 ± 13.4	86.6 ± 15.3	80.8 ± 14.9	74.3 ± 14.8	80.5 ± 15.8
Height (cm)	170.4 ± 10.0	172 ± 10.7	173.0 ± 9.4	168.4 ± 10.6	171.7 ± 9.8	175.3 ± 8.6	176.7 ± 8.5	171.8 ± 9.2	166.8 ± 10.1	171.8 ± 10.1
BMI (kg/m^2)	28.6 ± 4.9	27.1 ± 4.1	26.0 ± 3.7	27.1 ± 5	27.3 ± 4.6	27.3 ± 3.6	27.7 ± 4.1	27.4 ± 4.8	26.6 ± 4.3	27.2 ± 4.4
Waist circumference (cm)	100.9 ± 13.1	95.5 ± 12.6	91.9 ± 11.6	94.6 ± 14.1	95.2 ± 13.8	98.6 ± 10.4	99.8 ± 12.0	95.8 ± 13.4	92.8 ± 12.6	96.1 ± 13.0
Waist-to-hip ratio	1.0 ± 0.1	0.9 ± 0.1	0.9 ± 0.1	0.9 ± 0.1	0.9 ± 0.1	1.0 ± 0.1	1.0 ± 0.1	0.9 ± 0.1	0.9 ± 0.1	0.9 ± 0.1
Waist-to-height ratio	0.6 ± 0.1	0.6 ± 0.1	0.5 ± 0.1	0.6 ± 0.1	0.6 ± 0.1	0.6 ± 0.1	0.6 ± 0.1	0.6 ± 0.1	0.6 ± 0.1	0.6 ± 0.1
Systolic blood pressure (mmHg)	143.9 ± 18.6	138.8 ± 18.8	136.0 ± 16.1	138.3 ± 17.6	139.4 ± 18.7	141.0 ± 18.2	142.4 ± 17.7	136.9 ± 17.3	139.4 ± 19.1	139.6 ± 18.2
Diastolic blood pressure (mmHg)	86.5 ± 8.9	84.8 ± 8.5	83.9 ± 8.8	84.2 ± 8.4	85.2 ± 9.1	85.8 ± 8.8	86 ± 8.6	84.2 ± 9	85 ± 8.7	85.1 ± 8.8
Biochemical parameters										
Plasma C-reactive protein (mg/L)	5.6 ± 10.1	2.7 ± 2.7	2.9 ± 4.1	4.2 ± 6.1	4.5 ± 9.7	2.9 ± 3.3	4.6 ± 9.6	4.4 ± 6.7	2.8 ± 3.1	3.9 ± 7.0
Whole blood HbA1c (%)	5.9 ± 0.7	5.7 ± 0.5	5.6 ± 0.5	5.7 ± 0.7	5.7 ± 0.5	5.7 ± 0.6	5.7 ± 0.7	5.7 ± 0.6	5.7 ± 0.5	5.7 ± 0.6
Total plasma cholesterol (mg/dL)	224.6 ± 45.1	225.5 ± 39.4	222 ± 41.2	223.8 ± 42.7	225 ± 42.0	223.4 ± 41.2	216.5 ± 38.6	220.6 ± 44.8	235.1 ± 39.9	224.0 ± 41.9
Plasma HDL cholesterol (mg/dL)	61.8 ± 16.7	65.3 ± 18.3	70.4 ± 20.0	68.4 ± 20.2	67.3 ± 19.0	61.9 ± 16.1	61.2 ± 17.0	65.3 ± 18	71 ± 19.8	65.8 ± 18.7
Plasma LDL cholesterol (mg/dL)	133.9 ± 38.7	133.0 ± 30.3	128.6 ± 33.5	129.4 ± 34.6	131.3 ± 34.6	134.8 ± 33.7	129.6 ± 32.6	129.4 ± 36.7	136.6 ± 33.2	131.9 ± 34.3
Plasma triglycerides (mg/dL)	133.4 ± 74.9	121.7 ± 81.8	109.7 ± 67.8	120.2 ± 68.9	123.8 ± 81.8	120.6 ± 75.7	126.7 ± 78.9	117.3 ± 71.8	120.6 ± 75.9	121.6 ± 75.6
Dietary intake										
Energy intake (kcal)	2188 ± 577	2220.5 ± 590	2320 ± 601	2068 ± 570	2215 ± 551	2446 ± 592	2345 ± 564	2263 ± 610	2120 ± 579	2243 ± 591
Total carbohydrates (g/d)	218.5 ± 65.5	226.0 ± 72.4	230.5 ± 67.0	210.7 ± 68.9	222.7 ± 64.9	241.6 ± 68.3	231.8 ± 65.4	227.5 ± 74.6	215.7 ± 64.1	225.0 ± 68.4
Total protein (g/d)	79.1 ± 23.5	79.3 ± 23.6	80.8 ± 23.6	72.3 ± 21.6	79.6 ± 23.2	87.4 ± 23.3	84.2 ± 24.2	80.5 ± 22.5	74.6 ± 22.9	79.8 ± 23.5
Total fat (g/d)	97.3 ± 28	98 ± 28.5	103.6 ± 30.5	90.8 ± 27.2	98.8 ± 28.0	109.3 ± 29.3	105.8 ± 29.1	99.1 ± 27.7	94.0 ± 29.5	99.6 ± 29.1
n-6:n-3 ratio	5.9 ± 1.3	5.7 ± 1.2	5.6 ± 1.5	5.8 ± 1.5	5.8 ± 1.3	5.6 ± 1.2	5.9 ± 1.4	5.8 ± 1.3	5.5 ± 1.3	5.7 ± 1.3
Total fiber (g/d)	21.3 ± 5.8	22.1 ± 6.5	23.0 ± 6.4	21.1 ± 6.0	21.9 ± 6.4	23.5 ± 6.2	22.1 ± 6.4	22.1 ± 6.2	22.2 ± 6.2	22.1 ± 6.2
Alcohol (g/d)	14.4 ± 19.7	13.5 ± 15.9	17.3 ± 18.4	13.9 ± 18.4	13.5 ± 16.3	17.8 ± 19.2	15.4 ± 15.5	16.8 ± 20.9	13.0 ± 17.4	15.1 ± 18.1
Smoking status [*n* (%)]										
Never	99 (51)	92 (47)	100 (51)	99 (51)	103 (53)	89 (45)	90 (47)	91 (47)	110 (57)	291 (50)
Former	75 (39)	87 (45)	77 (40)	71 (37)	78 (40)	90 (46)	86 (44)	85 (43)	68 (35)	239 (41)
Current	20 (10)	16 (8)	17 (9)	24 (12)	14 (7)	15 (8)	18 (9)	19 (10)	16 (8)	53 (9)
Physical activities										
Total MET-h/wk	96.2 ± 55.3	111.2 ± 73.6	120.6 ± 70.6	86.4 ± 51.8	104.5 ± 59.3	137.1 ± 79.0	67.9 ± 37.2	104.8 ± 53.9	155.3 ± 74.9	109.3 ± 67.6
Housework (h/wk)	8.1 ± 7.3	8.4 ± 8.7	7.8 ± 8.5	8.1 ± 7.3	8.6 ± 8.4	7.6 ± 8.8	2.6 ± 3.0	6.0 ± 4.1	15.6 ± 9.2	8.1 ± 8.2
Walking (h/wk)	7.7 ± 7.8	6.8 ± 6.9	7.6 ± 7.7	6.7 ± 6.2	7.1 ± 7.6	8.3 ± 8.5	4.2 ± 3.9	7.6 ± 7.5	10.3 ± 8.8	7.4 ± 7.5
Gardening (h/wk)	4.0 ± 4.9	3.0 ± 5.1	2.4 ± 3.9	0.9 ± 2.6	2.9 ± 3.7	5.7 ± 5.9	2.5 ± 3.9	2.7 ± 3.7	4.2 ± 6.0	3.1 ± 4.7
Do-it-yourself work (h/wk)	2.4 ± 3.6	2.1 ± 3.2	1.4 ± 2.2	0.4 ± 0.9	1.5 ± 1.9	4.0 ± 4.2	2.1 ± 2.9	2.3 ± 3.4	1.6 ± 2.9	2.0 ± 3.1
Stair climbing (flights/d)	3.9 ± 4.7	4.2 ± 4.9	8.2 ± 8.8	3.1 ± 3.3	5.1 ± 6.0	8.2 ± 8.6	3.8 ± 4.4	6.0 ± 6.8	6.6 ± 8.0	5.5 ± 6.7
Sports (h/wk)	1.1 ± 2.5	3.2 ± 3.3	3.8 ± 3.3	2.8 ± 3.3	2.8 ± 3.5	2.5 ± 3.0	2.1 ± 2.8	2.9 ± 3.6	3.2 ± 3.4	2.7 ± 3.3
Cycling (h/wk)	1.2 ± 2.3	2.5 ± 3.1	4.0 ± 3.9	1.8 ± 3.0	2.1 ± 2.7	3.8 ± 3.9	1.8 ± 2.4	2.8 ± 3.4	3.2 ± 4.0	2.6 ± 3.4
MPA (h/wk)	22.3 ± 4.8	20.4 ± 4.8	19.5 ± 4.5	16.2 ± 3.4	20.3 ± 4.4	25.9 ± 5.5	11.5 ± 2.8	18.9 ± 3.8	31.9 ± 5.4	20.8 ± 4.8
VPA (h/wk)	2.3 ± 2.4	5.7 ± 3.2	7.8 ± 3.6	4.6 ± 3.2	4.9 ± 3.1	6.3 ± 3.5	3.9 ± 2.6	5.7 ± 3.5	6.4 ± 3.7	5.3 ± 3.4
Total activity reported (h/d)	3.5 ± 2.0	3.7 ± 2.6	3.9 ± 2.4	3.0 ± 1.8	3.6 ± 2.1	4.6 ± 2.7	2.2 ± 1.2	3.5 ± 1.8	5.5 ± 2.6	3.7 ± 2.3
Physical inactivities										
Sleeping (h/d)	8.2 ± 1.7	7.5 ± 1.0	7.0 ± 1.0	7.4 ± 1.2	7.4 ± 1.1	8.0 ± 1.6	7.1 ± 1.1	7.6 ± 1.1	8.0 ± 1.6	7.6 ± 1.3
Watching TV (h/d)	3.9 ± 2.4	3.0 ± 1.2	1.8 ± 1.0	2.8 ± 1.4	2.7 ± 1.7	3.1 ± 2.2	2.6 ± 1.4	2.8 ± 1.5	3.3 ± 2.4	2.9 ± 1.8
Total inactivity reported (h/d)	12.1 ± 3.3	10.5 ± 1.4	8.9 ± 1.2	10.2 ± 1.8	10.1 ± 2.3	11.1 ± 3.2	9.7 ± 1.7	10.4 ± 2.0	11.3 ± 3.3	10.5 ± 2.5
Overall 24-h inactivity (h/d)[b]	20.5 ± 2.0	20.3 ± 2.6	20.1 ± 2.4	21.0 ± 1.8	20.4 ± 2.1	19.4 ± 2.7	21.8 ± 1.2	20.5 ± 1.8	18.5 ± 2.6	20.3 ± 2.3
Volumes of adipose tissue[c]										
VAT (dm^3)	4.9 ± 2.2	4.0 ± 2.0	3.3 ± 1.8	3.8 ± 2.1	4.0 ± 2.3	4.4 ± 1.9	4.7 ± 2.2	4.1 ± 2.2	3.4 ± 1.8	4.1 ± 2.1
SAAT (dm^3)	7.9 ± 4.1	6.9 ± 3.2	6.0 ± 2.8	7.4 ± 4.1	7.1 ± 3.6	6.4 ± 2.5	6.7 ± 3.4	7.0 ± 3.9	7.2 ± 3.1	7.0 ± 3.5
STRAT (dm^3)[d]	11.3 ± 5.3	9.8 ± 4.2	8.6 ± 3.7	10.4 ± 5.4	10.1 ± 4.7	9.1 ± 3.3	9.5 ± 4.4	9.9 ± 5.2	10.2 ± 4.0	9.9 ± 4.6

[a] Data are means (± SD) or *n* (%). Abbreviations: BMI, body mass index (kg/m^2); n-6:n-3 ratio; ratio of n-6 to n-3 fatty acids; HbA1c, glycated hemoglobin A1c; MET-h, metabolic equivalent task hours per week (including housework, walking, gardening, do-it-yourself work, stair climbing, sports, cycling); MPA, medium-intensity physical activity; PA, physical activity; PCA, principal components analysis; SAAT, subcutaneous abdominal adipose tissue; STRAT, subcutaneous trunk adipose tissue; VAT, visceral abdominal adipose tissue; VPA, vigorous-intensity physical activity.

[b] Assumed overall 24-h inactivity was calculated as the difference between 24 hours and the sum of reported time (h/d) for all activities.

[c] Adipose tissue: 1 dm^3 = 1 L = ~0.9 kg.

[d] Subcutaneous trunk adipose tissue was measured from the humeral to the femoral heads.
