# Supplementary material for: Association of Habitual Patterns and Types of Physical Activity and Inactivity with MRI-Determined Total Volumes of Visceral and Subcutaneous Abdominal Adipose Tissue in a General White Population
Source: PLoS One. 2015 Nov 30;10(11):e0143925. doi: 10.1371/journal.pone.0143925 (PMC4664408; doi:10.1371/journal.pone.0143925)
Supplement: S2 Table — (PDF) [file pone.0143925.s002.pdf]

Supporting Information

"Association of habitual patterns and types of physical activity and inactivity with MRI-determined total volumes of visceral and subcutaneous abdominal adipose tissue in a general white population" *PLOS ONE*

Karina Fischer, Daniela Moewes, Hans-Peter Müller, Gunnar Jacobs, Jan Kassubek, Wolfgang Lieb, Ute Nöthlings

Corresponding author: Department of Geriatrics, University Hospital Zurich & Centre on Aging and Mobility, University of Zurich, Zurich, Switzerland. E-mail: karina.fischer@uzh.ch

S2 Table. Total volumes of visceral and subcutaneous abdominal adipose tissue for tertiles of PA MET, individual types of physical activity or inactivity, and activity patterns by sex in Northern German adults.[a]

Median (and range) of tertiles of total PA MET, PA, IA, and APAT	n [M/F]	VAT (dm^3)						SAAT (dm^3)					
		Men			Women			Men			Women		
		Unadjusted LSM ± SE	Adjusted LSM ± SE	(%)[b]	Unadjusted LSM ± SE	Adjusted LS mean ± SE	(%)[b]	Unadjusted LSM ± SE	Adjusted LSM ± SE	(%)[b]	Unadjusted LSM ± SE	Adjusted LSM ± SE	(%)[b]
**PA MET (h/wk)**													
51.5 (<74.1)	194 [138/56]	5.11 ± 0.18 ^{1} [c]	5.07 ± 0.18 ^{1}	100.0	3.05 ± 0.19 ^{1}	3.09 ± 0.19 ^{1}	100.0	6.54 ± 0.23 ^{1}	6.34 ± 0.23 ^{1}	100.0	9.26 ± 0.52 ^{1}	9.07 ± 0.52 ^{1}	100.0
94.8 (74.1-121.9)	195 [110/85]	5.15 ± 0.19 ^{1}	5.18 ± 0.19 ^{1}	102.2	2.93 ± 0.16 ^{1,2}	2.91 ± 0.16 ^{1,2}	94.2	6.09 ± 0.25 ^{1}	6.15 ± 0.24 ^{1}	87.1	8.22 ± 0.45 ^{1,2}	8.18 ± 0.45 ^{1,2}	90.2
155.6 (>121.9)	194 [94/100]	4.47 ± 0.21 ^{2}	**4.46 ± 0.21** ^{2} [d]	88.0	2.69 ± 0.15 ^{2}	**2.65 ± 0.15** ^{2}	85.8	5.55 ± 0.27 ^{2}	**5.63 ± 0.27** ^{2}	82.3	7.43 ± 0.41 ^{2}	**7.51 ± 0.41** ^{2}	82.8
	P linear trend		0.041			0.043			0.045			0.044	
	P quadratic trend		0.180			0.997			0.849			0.812	
**Types of PA**													
Housework (h/wk)													
2.0 (<3.0)	222 [194/28]	4.86 ± 0.15 ^{1}	4.89 ± 0.15 ^{1}	100.0	3.19 ± 0.28 ^{1}	3.35 ± 0.28 ^{1}	100.0	6.17 ± 0.19 ^{1}	6.11 ± 0.19 ^{1}	100.0	9.76 ± 0.77 ^{1}	9.52 ± 0.77 ^{1}	100.0
5.0 (3.0-9.0)	163 [100/63]	4.91 ± 0.21 ^{1}	4.88 ± 0.20 ^{1}	99.8	2.32 ± 0.18 ^{2}	**2.44 ± 0.19** ^{2}	72.8	5.90 ± 0.27 ^{1}	5.90 ± 0.26 ^{1}	96.6	7.38 ± 0.51 ^{2}	**7.26 ± 0.51** ^{2}	76.3
12.0 (>9.0)	198 [48/150]	5.34 ± 0.30 ^{1}	5.23 ± 0.29 ^{1}	107.0	3.03 ± 0.12 ^{1,2}	2.93 ± 0.12 ^{1,2}	87.5	6.33 ± 0.38 ^{1}	6.30 ± 0.37 ^{1}	103.1	8.20 ± 0.33 ^{1,2}	8.25 ± 0.34 ^{1,2}	86.7
	P linear trend		0.297			0.742			0.600			0.852	
	P quadratic trend		0.613			**0.002**[e]			0.616			**0.0006**	
DIY (h/wk)													
0.0 (0.0)	189 [49/140]	4.76 ± 0.30 ^{1}	4.82 ± 0.30 ^{1}	100.0	2.83 ± 0.13 ^{1}	2.75 ± 0.12 ^{1}	100.0	6.44 ± 0.38 ^{1}	6.24 ± 0.36 ^{1}	100.0	7.83 ± 0.34 ^{1}	7.83 ± 0.35 ^{1}	100.0
1.0 (0.0-1.5)	181 [111/70]	5.07 ± 0.20 ^{1}	5.04 ± 0.19 ^{1}	104.6	2.75 ± 0.18 ^{1}	2.83 ± 0.18 ^{1}	102.9	6.07 ± 0.25 ^{1}	6.00 ± 0.24 ^{1}	96.2	8.11 ± 0.49 ^{1,2}	8.03 ± 0.48 ^{1,2}	102.6
3.0 (>1.5)	213 [182/31]	4.92 ± 0.15 ^{1}	4.90 ± 0.15 ^{1}	101.7	3.24 ± 0.27 ^{1}	3.33 ± 0.26 ^{1}	121.1	6.05 ± 0.20 ^{1}	6.08 ± 0.19 ^{1}	97.4	9.79 ± 0.73 ^{2}	**9.79 ± 0.73** ^{2}	125.0
	P linear trend		0.961			0.062			0.682			0.020	
	P quadratic trend		0.457			0.866			0.755			0.588	
Gardening (h/wk)													
0.0 (<0.38)	186 [100/86]	4.98 ± 0.21 ^{1,2}	4.99 ± 0.20 ^{1,2}	100.0	2.87 ± 0.16 ^{1}	2.88 ± 0.16 ^{1}	100.0	5.88 ± 0.26 ^{1}	5.75 ± 0.25 ^{1}	100.0	8.62 ± 0.44 ^{1}	8.57 ± 0.44 ^{1}	100.0
1.3 (0.4-2.8)	198 [117/81]	4.47 ± 0.19 ^{1}	4.53 ± 0.19 ^{1}	90.8	2.69 ± 0.17 ^{1}	2.76 ± 0.16 ^{1}	95.8	5.84 ± 0.24 ^{1}	5.76 ± 0.24 ^{1}	100.2	7.49 ± 0.45 ^{1}	7.46 ± 0.46 ^{1}	87.0
6.0 (>2.8)	199 [125/74]	5.36 ± 0.18 ^{2}	5.27 ± 0.18 ^{2}	105.6	3.04 ± 0.17 ^{1}	2.91 ± 0.17 ^{1}	101.0	6.56 ± 0.24 ^{2}	**6.64 ± 0.23** ^{2}	115.5	8.37 ± 0.47 ^{1}	8.37 ± 0.48 ^{1}	97.7
	P linear trend		0.054			0.658			**0.001**			0.593	
	P quadratic trend		0.027			0.485			0.610			0.264	
**Walking (h/wk)**													
2.0 (<3.0)	185 [128/57]	4.99 ± 0.18 ^{1}	5.02 ± 0.18 ^{1}	100.0	3.30 ± 0.20 ^{1}	3.29 ± 0.19 ^{1}	100.0	6.38 ± 0.23 ^{1}	6.28 ± 0.23 ^{1}	100.0	9.65 ± 0.53 ^{1}	9.46 ± 0.53 ^{1}	100.0
5.0 (3.0-7.5)	202 [104/98]	5.05 ± 0.20 ^{1}	4.96 ± 0.20 ^{1}	98.8	2.75 ± 0.15 ^{1,2}	**2.72 ± 0.15** ^{2}	82.7	6.12 ± 0.26 ^{1}	6.07 ± 0.25 ^{1}	96.7	7.62 ± 0.41 ^{2}	**7.59 ± 0.41** ^{2}	80.2
11.5 (>7.5)	196 [110/86]	4.80 ± 0.20 ^{1}	4.80 ± 0.20 ^{1}	95.6	2.70 ± 0.16 ^{2}	**2.71 ± 0.16** ^{2}	82.4	5.80 ± 0.25 ^{1}	5.85 ± 0.25 ^{1}	93.2	7.80 ± 0.43 ^{2}	**7.85 ± 0.44** ^{2}	83.0
	P linear trend		0.433			0.034			0.260			0.058	
	P quadratic trend		0.943			0.148			0.632			0.138	
Stair climbing (stairs/d)													
1.0 (<1.0)	154 [91/63]	5.19 ± 0.22 ^{1,2}	5.16 ± 0.21 ^{1,2}	100.0	2.93 ± 0.19 ^{1}	2.87 ± 0.18 ^{1}	100.0	6.38 ± 0.28 ^{1}	6.36 ± 0.27 ^{1}	100.0	8.27 ± 0.52 ^{1}	8.15 ± 0.52 ^{1}	100.0
3.0 (1.0-4.0)	224 [120/104]	5.29 ± 0.19 ^{1}	5.22 ± 0.19 ^{1}	101.2	2.97 ± 0.15 ^{1}	2.97 ± 0.14 ^{1}	103.5	6.37 ± 0.24 ^{1}	6.19 ± 0.24 ^{1}	97.3	8.47 ± 0.40 ^{1}	8.46 ± 0.40 ^{1}	103.8
10.0 (>4.0)	205 [131/74]	4.46 ± 0.18 ^{2}	**4.51 ± 0.18** ^{2}	87.4	2.65 ± 0.17 ^{1}	2.67 ± 0.17 ^{1}	93.0	5.70 ± 0.23 ^{1}	5.78 ± 0.23 ^{1}	90.9	7.64 ± 0.48 ^{1}	7.67 ± 0.47 ^{1}	94.1
	P linear trend		**0.005**			0.346			0.145			0.265	
	P quadratic trend		0.375			0.449			0.787			0.391	
Cycling (h/wk)													
0.0 (<0.4)	186 [100/86]	5.80 ± 0.20 ^{1}	5.72 ± 0.20 ^{1}	100.0	3.39 ± 0.16 ^{1}	3.29 ± 0.16 ^{1}	100.0	7.21 ± 0.26 ^{1}	7.16 ± 0.25 ^{1}	100.0	9.19 ± 0.44 ^{1}	9.21 ± 0.44 ^{1}	100.0
1.3 (0.4-2.6)	203 [122/81]	4.69 ± 0.18 ^{2}	**4.73 ± 0.18** ^{2}	82.7	2.64 ± 0.16 ^{2}	**2.69 ± 0.16** ^{2}	81.8	5.91 ± 0.23 ^{2}	**5.85 ± 0.23** ^{2}	81.7	7.62 ± 0.45 ^{1}	**7.56 ± 0.45** ^{2}	82.1
5.0 (>2.6)	194 [120/74]	4.49 ± 0.18 ^{2}	**4.49 ± 0.18** ^{2}	78.5	2.50 ± 0.17 ^{2}	**2.51 ± 0.17** ^{2}	76.3	5.41 ± 0.23 ^{2}	**5.41 ± 0.23** ^{2}	75.6	7.57 ± 0.47 ^{1}	**7.52 ± 0.47** ^{2}	81.7
	P linear trend		**0.0001**			**0.004**			**<0.0001**			0.064	
	P quadratic trend		**0.016**			0.070			0.070			0.344	
Sports (h/wk)													
0.0 (<0.9)	182 [116/66]	5.28 ± 0.19 ^{1}	5.24 ± 0.19 ^{1}	100.0	3.33 ± 0.18 ^{1}	3.22 ± 0.18 ^{1}	100.0	6.55 ± 0.24 ^{1}	6.53 ± 0.24 ^{1}	100.0	8.90 ± 0.50 ^{1}	8.94 ± 0.51 ^{1}	100.0
2.5 (0.9-3.0)	231 [121/110]	4.95 ± 0.19 ^{1,2}	4.92 ± 0.18 ^{1,2}	93.9	2.77 ± 0.14 ^{1,2}	2.78 ± 0.14 ^{1,2}	86.3	6.15 ± 0.24 ^{1,2}	6.11 ± 0.23 ^{1,2}	93.6	8.09 ± 0.39 ^{1}	8.06 ± 0.39 ^{1}	90.2
5.0 (>3.0)	170 [105/65]	4.57 ± 0.20 ^{2}	**4.61 ± 0.20** ^{2}	88.0	2.54 ± 0.18 ^{2}	**2.60 ± 0.18** ^{2}	80.7	5.58 ± 0.26 ^{2}	**5.54 ± 0.25** ^{2}	84.8	7.54 ± 0.51 ^{1}	7.43 ± 0.51 ^{1}	83.1
	P linear trend		**0.013**			**0.013**			**0.001**			0.084	
	P quadratic trend		0.958			0.345			0.785			0.967	
**Types of IA**													
Sleep (h/d)													
7.0 (<7.3)	250 [142/ 108]	4.99 ± 0.17 ^{1}	5.06 ± 0.15 ^{1}	100.0	2.59 ± 0.14 ^{1}	2.59 ± 0.14 ^{1}	100.0	6.00 ± 0.22 ^{1}	5.92 ± 0.22 ^{1}	100.0	7.62 ± 0.39 ^{1}	7.65 ± 0.39 ^{1}	100.0
8.0 (7.3-8.2)	209 [127/82]	4.70 ± 0.18 ^{1}	**4.68 ± 0.18** ^{2}	92.5	3.13 ± 0.16 ^{2}	**3.06 ± 0.16** ^{2}	118.1	6.16 ± 0.24 ^{1}	6.17 ± 0.23 ^{1}	104.2	8.47 ± 0.45 ^{1}	8.44 ± 0.45 ^{1}	110.3
9.0 (>8.2)	124 [73/51]	5.29 ± 0.24 ^{1}	5.12 ± 0.23 ^{1}	101.2	3.01 ± 0.21 ^{1,2}	3.07 ± 0.20 ^{1,2}	118.5	6.26 ± 0.31 ^{1}	6.23 ± 0.30 ^{1}	105.2	8.83 ± 0.57 ^{1}	8.71 ± 0.58 ^{1}	113.9
	P linear trend		0.820			0.025			0.229			0.037	
	P quadratic trend		0.082			0.200			0.861			0.642	
Watching TV (h/d)													
2.0 (<2.0)	253 [145/108]	4.48 ± 0.17 ^{1}	4.54 ± 0.17 ^{1}	100.0	2.51 ± 0.14 ^{1}	2.57 ± 0.14 ^{1}	100.0	5.74 ± 0.22 ^{1}	5.64 ± 0.21 ^{1}	100.0	7.56 ± 0.39 ^{1}	7.29 ± 0.39 ^{1}	100.0
3.0 (2.0-3.0)	166 [95/71]	4.79 ± 0.21 ^{1}	4.77 ± 0.20 ^{1}	105.1	2.90 ± 0.17 ^{1,2}	2.88 ± 0.17 ^{1,2}	112.1	5.86 ± 0.27 ^{1}	5.82 ± 0.26 ^{1}	103.2	8.18 ± 0.48 ^{1,2}	8.21 ± 0.48 ^{1,2}	112.6
4.0 (>3.0)	164 [102/62]	5.74 ± 0.20 ^{2}	**5.65 ± 0.20** ^{2}	124.4	3.43 ± 0.18 ^{2}	**3.29 ± 0.19** ^{2}	128.0	6.87 ± 0.26 ^{2}	**6.94 ± 0.26** ^{2}	123.0	9.21 ± 0.52 ^{2}	**9.53 ± 0.54** ^{2}	130.7
	P linear trend		**<0.0001**			**0.0007**			**0.0001**			**0.0006**	
	P quadratic trend		0.250			0.840			0.090			0.938	
**Activity patterns**													
APAT-1													
-1.01 (<-0.33)	194 [114/80]	5.73 ± 0.19 ^{1}	5.67 ± 0.19 ^{1}	100.0	3.63 ± 0.16 ^{1}	3.54 ± 0.16 ^{1}	100.0	6.91 ± 0.24 ^{1}	7.01 ± 0.24 ^{1}	100.0	9.42 ± 0.45 ^{1}	9.57 ± 0.46 ^{1}	100.0
0.17 (-0.33-0.54)	195 [116/79]	4.99 ± 0.18 ^{2}	**4.97 ± 0.18** ^{2}	87.7	2.64 ± 0.16 ^{2}	**2.64 ± 0.16** ^{2}	74.6	6.18 ± 0.24 ^{1}	**6.09 ± 0.23** ^{2}	86.9	8.02 ± 0.45 ^{1,2}	**7.91 ± 0.44** ^{2}	82.7
0.85 (>0.54)	194 [142/108]	4.09 ± 0.19 ^{3}	**4.15 ± 0.19** ^{3}	73.2	2.32 ± 0.15 ^{2}	**2.39 ± 0.16** ^{3}	67.5	5.23 ± 0.24 ^{2}	**5.12 ± 0.23** ^{3}	73.0	7.07 ± 0.44 ^{2}	**6.96 ± 0.44** ^{2}	72.7
	P linear trend		**<0.0001**			**<0.0001**			**<0.0001**			**0.0005**	
	P quadratic trend		0.149			0.409			0.107			0.999	
APAT-2													
-0.90 (<-0.48)	194 [112/82]	5.11 ± 0.24 ^{1}	5.08 ± 0.23 ^{1}	100.0	2.92 ± 0.14 ^{1}	2.89 ± 0.14 ^{1}	100.0	6.03 ± 0.30 ^{1}	5.84 ± 0.29 ^{1}	100.0	8.25 ± 0.38 ^{1}	8.23 ± 0.38 ^{1}	100.0
0.44 (-0.48 - 0.64)	195 [107/88]	5.06 ± 0.20 ^{1}	5.12 ± 0.20 ^{1}	100.8	2.78 ± 0.16 ^{1}	2.79 ± 0.16 ^{1}	96.5	6.33 ± 0.26 ^{1}	6.34 ± 0.25 ^{1}	108.6	8.12 ± 0.44 ^{1}	8.04 ± 0.43 ^{1}	97.7
1.01 (>0.64)	194 [158/36]	4.78 ± 0.17 ^{1}	4.74 ± 0.16 ^{1}	93.3	2.87 ± 0.25 ^{1}	2.88 ± 0.25 ^{1}	99.7	6.01 ± 0.21 ^{1}	6.02 ± 0.21 ^{1}	103.1	7.99 ± 0.69 ^{1}	8.07 ± 0.70 ^{1}	98.1
	P linear trend		0.362			0.987			0.282			0.425	
	P quadratic trend		0.376			0.487			0.765			0.717	
APAT-3													
-0.84 (<-0.42)	194 [77/117]	4.99 ± 0.16 ^{1}	4.98 ± 0.16 ^{1}	100.0	2.58 ± 0.30 ^{1}	2.74 ± 0.30 ^{1}	100.0	6.29 ± 0.20 ^{1}	6.19 ± 0.20 ^{1}	100.0	9.17 ± 0.81 ^{1}	8.99 ± 0.81 ^{1}	100.0
-0.04 (-0.42 - 0.32)	195 [107/88]	4.83 ± 0.19 ^{1}	4.85 ± 0.19 ^{1}	97.4	3.06 ± 0.17 ^{1}	3.17 ± 0.17 ^{1}	115.7	5.69 ± 0.24 ^{1}	5.74 ± 0.24 ^{1}	92.7	9.05 ± 0.46 ^{1}	8.99 ± 0.47 ^{1}	100.0
0.58 (>0.32)	194 [158/36]	5.06 ± 0.28 ^{1}	4.96 ± 0.28 ^{1}	99.6	2.81 ± 0.13 ^{1}	2.69 ± 0.13 ^{1}	98.2	6.48 ± 0.36 ^{1}	6.46 ± 0.35 ^{1}	104.4	7.50 ± 0.34 ^{1}	**7.52 ± 0.35** ^{2}	83.6
	P linear trend		0.821			0.185			0.874			0.030	
	P quadratic trend		0.444			0.106			0.040			0.440	

[a] Data (*n* = 583 subjects) are unadjusted or adjusted LSM (± SE) of total volumes of VAT or SAAT from generalized linear models adjusted for age, total energy intake (kcal/d), smoking status (never, former, and current smoker), and height (final model 2) by sex and tertile category of individual activity or APAT variables (see also Fig 3 and Fig 1). Multiple comparisons assessing statistical differences in unadjusted or adjusted LSM values between tertiles were corrected by using the Tukey-Kramer procedure. Linear regression analysis was used to test for a linear or quadratic trend across the tertiles by treating the median values of each category as continuous variables. P_{trend} indicates *P* for a linear or quadratic trend across median values of non-sex-specific tertile categories of activity variables. *P* values are two-sided and uncorrected. The B-Y FDR threshold *P* value for 13 possibly dependent tests is 0.016. Bolded LSM or *P* values indicate a statistical significance at the B-Y FDR level. Abbreviations: APAT 1-3; activity pattern 1 to 3 derived by principal components analysis; BMI, body mass index (kg/m^2); B-Y FDR, Benjamini-Yekutieli false discovery rate; DIY, do-it-yourself; F, females; IA, physical inactivity; LSM, least-square means; M, males; PA, physical activity; PA MET, metabolic equivalent hours per week of total physical activity (including housework, walking, gardening, do-it-yourself work, stair climbing, sports, cycling); SAAT, subcutaneous abdominal adipose tissue; VAT, visceral abdominal adipose tissue.

[b] All such values: Percentage (%) of adjusted LSM values compared to the first tertile (100%).

[c] Post-hoc comparisons between tertiles were corrected by using the Tukey-Kramer procedure: Mean values without sharing a common superscript number (1-3) were statistically different at $P < 0.05$.

[d] All such bolded LSM values: Statistically different compared to the reference category (first tertile) at B-Y FDR level ($P \leq 0.016$).

[e] All such bolded P_{trend} values: Statistically significant at B-Y FDR level ($P \leq 0.016$).
